# Enhanced cognitive control following neurofeedback therapy in chronic treatment-resistant PTSD among refugees: a feasibility study

**DOI:** 10.3389/fpsyt.2025.1567809

**Published:** 2025-08-15

**Authors:** Mirjana Askovic, Sejla Murdoch, René Mayer-Pelinski, Anna J. Watters, James Elhindi, Jorge Aroche, Juri D. Kropotov, Anthony W.F. Harris

**Affiliations:** ^1^ Neurofeedback Services, New South Wales Service for the Treatment and Rehabilitation of Torture and Trauma Survivors (STARTTS), Sydney, NSW, Australia; ^2^ Specialty of Psychiatry, Sydney Medical School, University of Sydney, Westmead, NSW, Australia; ^3^ Brain Dynamics Centre, Westmead Institute for Medical Research, Westmead, NSW, Australia; ^4^ Practice for Psychotherapy, Ottendorf-Okrilla, Germany; ^5^ Research & Development, BEE Medic GmbH, Singen, Germany; ^6^ Research and Education Network, Western Sydney Local Health District, Sydney, NSW, Australia; ^7^ Natalia Petrovna (N.P.) Bechtereva Institute of the Human Brain, Russian Academy of Sciences, Saint-Petersburg, Russia

**Keywords:** neurofeedback, refugee trauma, chronic post-traumatic stress disorder, event-related potentials, cognitive control, neuromarkers

## Abstract

**Background:**

Post-traumatic stress disorder (PTSD) is a debilitating condition affecting 3.9% of the global population, with refugee populations experiencing particularly high prevalence rates (23–42%). Cognitive control deficits are a core feature of PTSD and a significant factor in treatment resistance, which affects 25–60% of cases.

**Methods:**

This study examined the effects of neurofeedback therapy (NFT) on PTSD symptoms and cognitive control in forty-seven refugees with chronic treatment-resistant PTSD. Pre- and post-treatment assessments included the Harvard Trauma Questionnaire (HTQ), event-related potential (ERP) and behavioural parameters recorded during a cued Go/No-Go task. Over a median of twenty-six sessions across 7 months, clients received individualised NFT integrated with trauma counselling. Post-treatment, clients were categorised into *Responders* and *Non-Responders*, with responders defined as those achieving a clinically significant reduction in PTSD symptoms (≥0.5-point decrease on the HTQ).

**Results:**

Responders (n=22) demonstrated normalised P3d amplitude, indicative of improved cognitive control. In contrast, non-responders (n=25) exhibited minimal changes in ERP measures. Non-responders showed greater abnormalities in the Slow Positive Wave (SPW) at baseline suggesting more compromised late-stage cognitive processing.

**Discussion:**

These findings suggest that NFT can alleviate PTSD symptoms in refugees with chronic treatment-resistant PTSD. Treatment response was associated with a normalisation of the P3d waveform suggestive of enhanced cognitive control. The baseline SPW predicted treatment response. Further research should incorporate randomised controlled trials and larger, multi-centre samples to enhance robustness and generalisability.

## Introduction

Post-traumatic stress disorder (PTSD) is a widespread and debilitating mental health condition, affecting an estimated 3.9% of the global population (World Health Organization (1). Defined by symptoms such as intrusive memories, avoidance behaviours, negative shifts in cognition and mood, and altered arousal, PTSD disrupts daily functioning and diminishes quality of life (2). Refugee populations, due to experiences of war, torture, forced displacement, and profound loss, exhibit particularly high rates of PTSD, ranging from 23% to 42% (3, 4). Although evidence-based interventions, including trauma-focussed psychotherapy and pharmacotherapy, have positive results, 25% to 60% of those exposed to severe trauma experience treatment-resistant PTSD (5, 6).

Cognitive control deficits are one of the core features of PTSD (7) and a significant factor in treatment resistance (8). These impairments, encompassing difficulties with attention, working memory, response inhibition, conflict monitoring, and adaptive response modulation, impact the ability to regulate emotions, suppress automatic responses to trauma-related cues, and engage in goal-directed behaviour (e.g., 8–10).

These neural dysfunctions are reflected in alterations in the brain electrical activity, measured by the event-related potentials (ERPs) to provide precise temporal insights into cognitive processing. Studies employing paradigms such as Go/No-Go and Oddball tasks have revealed specific ERP alterations, including deviations in P3, and the Late Positive Potential (LPP).

The P3, a positive-going event-related potential, occurs approximately three hundred milliseconds after the presentation of a stimulus and reflects cognitive processes related to attention and working memory. It has two main subcomponents: P3a (frontal), associated with orienting to novel stimuli and P3b (parietal), linked to context updating and working memory (11, 12). An additional component, P3d (frontal), is observed in No-Go trials and is associated with response inhibition (13, 14).

Research consistently demonstrates disrupted P3 component activity in PTSD. Prolonged P3 latencies for trauma-related stimuli indicate delayed decision-making processes (15). This has been associated with higher hyperarousal scores (16). Studies using visual Oddball task with threat-related distractors revealed an increased P3 amplitude to both standard stimuli and trauma-related distractors, indicating heightened attentional focus on both trauma-related and neutral, irrelevant information, along with difficulties in sustained attention and working memory, suggesting a hypersensitive salience detection network in PTSD (17). For paradigms containing non-affective or neutral stimuli, P3 amplitude was observed to be smaller in clients with PTSD compared to normal controls (12). Reduced P3 amplitudes may suggest diminished attentional capacity, and therefore difficult for individuals to allocate attention to non-threatening, novel information (18). A reduced P3 response to task-relevant information showed the strongest correlation with numbing and avoidance symptoms, suggesting that individuals with PTSD may adopt a strategy to limit attentional resources and minimise emotional arousal (19). Task dependent differences in the P3 amplitude suggest that PTSD involves a context-dependent disruption in information processing (18).

The slow positive wave (SPW) is a positive-going event-related potential (ERP) component that occurs within 500–1000 ms post-stimulus. Although temporally overlapping with the P3b, the SPW exhibits distinct functional properties (20). Unlike the transient P3b, the SPW demonstrates a longer enhancement lasting several seconds following motivationally salient stimuli (20, 21). This prolonged neural activity reflects sustained attention and cognitive effort, particularly during working memory maintenance and cognitively demanding tasks (22).

The SPW corresponds to the late positive potential (LPP) referred to in the emotion research (23). The LPP, a marker of sustained attention to emotionally loaded stimuli, is modulated by stimulus significance. Elevated LPP amplitudes to negative stimuli have been linked to heightened fear and anxiety (24), while reduced amplitudes to positive stimuli could be associated with decreased responsiveness to positive environment and heightened vulnerability to depressive symptoms (25). Acute stress impairs the LPP's ability to differentiate between emotional and neutral stimuli over time. Alomari et al. (21) demonstrated an increased LPP response to neutral stimuli 30–40 minutes after stress induction, suggesting that heightened emotional arousal can lead to misinterpretation of neutral stimuli as emotionally salient. This stress-induced prolonged LPP reflects an ongoing state of emotional dysregulation, where the brain struggles to "switch off" emotional responses, even in the absence of emotionally evocative stimuli (21).

Individuals with PTSD show alterations in LPP activity, with some studies reporting enhanced LPP amplitudes, suggesting hypervigilance and heightened emotional reactivity (26) while others report blunted LPP responses to threat, indicative of reduced emotional response or emotional numbing (27). These conflicting findings indicate the heterogeneity of PTSD and could represent the dissociative subtype characterised by significant emotional numbing (28) and highlight the importance of considering individual differences in symptom presentation and neural responses.

While the relationship between the SPW and PTSD has not been directly investigated, research points to abnormalities in late ERP waves in individuals with this disorder. For instance, Weber et al. (29) found reduced positive wave over frontal and parietal areas (400-800ms) during a working memory updating task in individuals with PTSD compared to controls. This suggests that PTSD may affect sustained cognitive processes reflected in these later ERP components.

These electrophysiological findings emphasise the necessity of targeted interventions to address these deficits (30) and open an avenue for evaluating targeted brain-based training interventions.

Neurofeedback is a non-invasive therapeutic approach that uses electroencephalographic (EEG) sensors to monitor real-time brain activity, providing feedback to individuals so they can learn to modulate abnormal brainwave patterns through operant conditioning. By targeting specific brainwave frequencies, neurofeedback has the potential to improve both cognitive and emotional control (31–33). A recent meta-analysis of 17 randomised controlled trials—13 of which used EEG-based neurofeedback—confirmed that neurofeedback significantly reduces PTSD symptoms, with clinically meaningful improvements observed across standardised measures such as the CAPS-5 and PCL-5 (34). Given the well-documented cognitive and neural processing deficits associated with PTSD, neurofeedback emerges as a particularly promising intervention. For instance, Shaw et al. (28) demonstrated that alpha-down neurofeedback enhanced engagement of top-down emotional and cognitive control centres, which are often impaired in PTSD. In refugee populations, our previous proof of concept studies (35, 36) have shown that neurofeedback improves cognitive control and attentional focus, as demonstrated in normalisation of the P3 amplitude, while simultaneously reducing PTSD symptoms in refugees after NFT.

### Study aims

This study explores the efficacy of neurofeedback integrated with trauma counselling (NFT) in enhancing cognitive control and reducing PTSD symptoms among refugees with chronic treatment-resistant PTSD. Building on our previous research, we will examine the difference between P3 No-Go and P3 Go amplitudes in a cued Go/No-Go task as a neurophysiological marker of cognitive control. Participants will be classified into two groups—Responders and Non-Responders—based on changes in PTSD symptoms. The study aims to identify neurophysiological markers of treatment response and predictors of NFT efficacy.

### Hypotheses

#### Primary hypotheses

1. Individuals with chronic treatment-resistant PTSD will exhibit reduced P3d amplitude during a cued Go/No-Go task, reflecting deficits in reactive cognitive control, compared to a normative healthy control group. This aligns with prior research highlighting impaired cognitive control in PTSD.
2. NFT will increase P3 amplitude, indicative of enhanced cognitive control. This improvement is expected to be associated with a significant reduction in PTSD symptom severity.
3. An additional *exploratory aim* is to investigate whether baseline PTSD-related ERP features, such as the amplitude of the P3d and slow positive wave, can predict the response to NFT.

## Materials and methods

### Study design

The study used a retrospective, one-arm, pre-post design, based on collected data from adult clients treated at the Neurofeedback Clinic at the NSW Service for the Treatment and Rehabilitation of Torture and Trauma Survivors (STARTTS) in Sydney, Australia. The study has been retrospectively reviewed against the CRED-nf checklist (37) for reporting standards in neurofeedback research. A completed checklist is provided as Supplementary Material (**Supplementary Table 3**).

### Clients

All clients participated in an ongoing neurofeedback program for chronic PTSD associated with refugee experiences. They received treatment at the Service for the Treatment and Rehabilitation of Torture and Trauma Survivors (STARTTS) in South-West Sydney, Australia, between February 2017 and October 2020. The clients referred to the neurofeedback program, had not responded to previous trauma counselling and pharmacotherapy, which were the first-line interventions, and had been consequently referred for NFT.

All clients met DSM-5 diagnostic criteria for PTSD, had a history of refugee-related trauma, had completed a minimum of ten sessions of NFT and were aged 21 years or older. Exclusion criteria included prior treatment with NFT, confirmed severe traumatic brain injury that required hospitalisation, neurological disorders, substance-related disorders (excluding nicotine and caffeine), and the need for urgent medical treatment. This cohort represented a subset of clients from a broader study examining neurofeedback outcomes for refugee-related PTSD, conducted at STARTTS. Some of the participant data for this study (34 clients) were drawn from clients described in the following papers: Askovic et al. (36) and Askovic et al (35, 38). An additional thirteen clients' data were collected for this study and have not been included in any other research.

This study was designed as a retrospective feasibility study, and as such, a formal *a priori* power calculation was not conducted. The sample size was determined by the number of eligible clinical cases meeting the inclusion criteria during the study period. While this limits the ability to draw definitive conclusions about effect size or generalisability, the sample was sufficient for exploratory ERP and behavioural comparisons. The need for future prospective studies with powered sample size calculations is acknowledged in the limitations. This approach is consistent with current recommendations for sample size reporting in exploratory neuroimaging research (39).

A control group consisting of 107 healthy individuals, matched for age, was drawn from the Human Brain Index (HBI) database (40, 41) for comparison of ERP and behavioural data.

### Treatment procedure

The treatment protocol consisted of one-hour sessions held one to two times per week. Each session included 10 to 20 minutes of neurofeedback (NF), along with time set aside for trauma counselling to address both the physiological and psychological components of refugee trauma. Symptom changes and responses to neurofeedback were recorded in the client’s medical records.

NF was delivered using the EEGer-4 software with J & J Spectrum 4 channel amplifier - J 404. The EEGer system used in this study provided simultaneous visual and auditory feedback linked to participants’ ongoing EEG signals. Most clients interacted with the *Formation* game, which progressively revealed full images—such as nature scenes, animals, or photos chosen by the clients themselves—as they met reinforcement criteria. This format supported both cultural sensitivity and personal relevance. When a participant found a particular set distressing or disengaging, it was replaced with an alternative. Simple tones were also used to reinforce successful regulation and support sustained attention. The reinforcement threshold was typically maintained between 60% and 70%, with real-time adjustments made by the clinician to optimise training effectiveness and comfort. Clinicians initially set NF protocols—including electrode placement and training frequencies—tailored to the client’s symptoms of over- or under-arousal (42) and arousal stability (43, 44). The most common neurofeedback protocols involved enhancing high alpha activity (9–13 Hz) at the parietal and temporal sites, often combined with enhancing low beta/reducing lower alpha activity (8–10 Hz) in the central and frontal sites (See **Table 1**). These protocols were adjusted dynamically based on the client's feedback and the therapist's observations during each session. A typical session began by gathering verbal feedback from the client, focussing on any changes in symptoms, especially in terms of arousal levels and stability. These reports were monitored alongside observable shifts in behaviour or functioning since the previous session. The NF training lasted between 10 and 20 minutes and it was integrated into one hour trauma counselling. The counselling addressed the biopsychosocial aspects of refugee experiences, using a culturally sensitive approach to address both past traumas and resettlement challenges (45).

**Table 1:** Baseline characteristics of responders and non-responders.

Comparison	Responders (N=22)	Non-responders (N=25)	Statistical test	P-value
Gender (Male/Female)	10M/12F	15M/10F	χ² = 0.50	0.48
Age (years)	45.5 (41–53)	50 (41–57)	U = 246.0	0.54
Residential Status: T- temporary P-permanent	T = 4 (18%) P = 18(82%)	T= 3 (12%) P = 22 (88%)	χ² = 0.03	0.85
Years of Education	12 (8–15)	12 (8–16)	U = 284.0	0.85
Living Alone	8 (36%)	7 (28%)	χ² = 0.09	0.76
Use of Interpreter	19 (86%)	18 (72%)	χ² = 0.71	0.40
Use of Psychotropic Medication ^a^	18 (82%)	13 (52%)	χ² = 3.40	0.065
Number of Medications	1 (1–2)	2 (1–2)	U = 86.5	0.186
Early Trauma	9 (41%)	7 (28%)	χ² = 0.39	0.53
Exposure to Torture ^b^	11 (50%)	8 (32%)	χ² = 0.92	0.34
Suspected Head Injury	10 (45%)	8 (32%)	χ² = 0.42	0.52
HTQ Score	3.2 (3–3.4)	2.6 (2.2–2.8)	U = 474.5	p < 0.001*
Total counselling sessions before NFT	27.5 (14.2-48.2)	23 (13-41)	U=258	p=0.726
Months in NFT	8.5 (6-15)	7 (5-10)	U = 218.5	p= 0.234
Number of NF sessions	29 (21.5-44.5)	25 (20-37)	U = 221.5	p=0.258

Baseline comparison of demographic and clinical factors between responders and non-responders. Responders were defined as participants with a decrease in Harvard Trauma Questionnaire (HTQ) score of 0.5 or greater from baseline to follow-up. Statistical significance was assessed using Chi-square or Mann-Whitney U tests, as appropriate. Significant results (p < 0.05) are highlighted with an asterisk (*).

^a^ Medications used by participants: Analgesics (including opioids, n = 9); Anticonvulsants (n = 5); Selective serotonin reuptake inhibitors (SSRIs, n = 12); Serotonin-norepinephrine reuptake inhibitors (SNRIs, n = 6); Other antidepressants (e.g., mirtazapine and bupropion, n = 5); Antipsychotics (n = 6); Tricyclic antidepressants (n = 9); Anti-anxiety agents (n = 4); Stimulants (n = 1).

^b^ Other traumas: Witnessing atrocities or the violent death of a family member (89%); Other refugee-related traumas such as dangerous flight, targeted harassment, forced separation, communal violence, deprivation, and destruction of home (98%).

### Assessment procedures

As part of standard clinical procedures at STARTTS, all clients underwent a detailed clinical assessment focussed on clinical symptoms, trauma history, and current psychosocial stressors. Sociodemographic data, type of refugee-related trauma (e.g., combat, imprisonment, torture), and early life trauma was extracted from the clinical notes (**Table 2**). Clinical data (such as use of psychotropic medication and history of brain injury) was obtained during the EEG assessment. Client’s also completed the Harvard Trauma Questionnaire, while event-related potential (ERP) and behavioural parameters were recorded during a cued Go/No-Go task, both pre- and post-treatment. This data was reviewed retrospectively. Clients gave their informed consent for the use of their data in clinical research during their initial EEG evaluation.

**Table 2:** Baseline demographic and clinical characteristics (n=47).

Comparison	PTSD group total (N=47)
Gender (Male/Female)	47% Female
Age (years)	47 (41–54.5)
Residential status:	
T- temporary P-permanent	T= 7 (15%) P = 40 (85%)
Years of Education	12 (8–16)
Living Alone	15 (32%)
Use of Interpreter	37 (79%)
Use of Psychotropic Medication ^a^	31 (66%)
Number of Medications	1 (1–2.5)
Early Trauma	16 (34%)
Exposure to Torture ^b^	19 (40%)
Suspected Head Injury	18 (38%)
HTQ Score ^c^	2.9 (2.5–3.2)
HSCL-Anxiety Score ^d^	2.7 (2.3-3.2)
HSCL-Depression Score ^e^	2.8 (2.5-3.3)
Total counselling sessions before NFT	24 (13.5-46)
Months in NFT	7 (5.5-12)
Number of NF sessions	26 (20.5-38.5)

^a^Psychotropic medications used by clients included analgesics (including opioids, *n* = 9), anticonvulsants (*n* = 5), selective serotonin reuptake inhibitors (SSRIs, *n* = 12), serotonin-norepinephrine reuptake inhibitors (SNRIs, *n* = 6), other antidepressants (e.g., mirtazapine and bupropion, *n* = 5), antipsychotics (*n* = 6), tricyclic antidepressants (*n* = 9), anxiolytics (*n* = 4), and stimulants (*n* = 1). Some clients were prescribed multiple medications.

^b^Other trauma exposures included witnessing atrocities or the violent death of a family member (89%), as well as other refugee-related experiences such as dangerous flight, targeted harassment, forced separation, communal violence, deprivation, and destruction of home (98%).

^c^HTQ – Harvard Trauma Questionnaire (cut-off score = 2.5).

^d^HSCL-Anxiety – Hopkins Symptom Checklist – Anxiety subscale (cut-off score = 1.75).

^e^HSCL-Depression – Hopkins Symptom Checklist – Depression subscale (cut-off score = 1.75).

This study was approved by the South Western Sydney Local Health District Human Research Ethics Committee (HREC NO. LNR/15/LPOOL/369).

### Measures

#### Clinical outcome measures

PTSD symptoms were evaluated using the Harvard Trauma Questionnaire (HTQ), version 4 (46), pre and post treatment. To minimise language barrier, the questionnaires were administered via the standardised multilingual computer-assisted platform (MultiCASI), which presented items both in written and spoken forms in relevant languages (47).

#### Cued Go/No-Go task/ Visual Continuous Performance Task

Clients completed the cued Go/No-Go task to assess event-related potentials (ERPs) associated with cognitive control and simultaneous behavioural performance (40, 41) both before and after completing NFT. ERP signals were recorded for both Go and No-Go conditions. The task was designed to evaluate attention, response inhibition, and reaction time (41).

**Figure 1:**
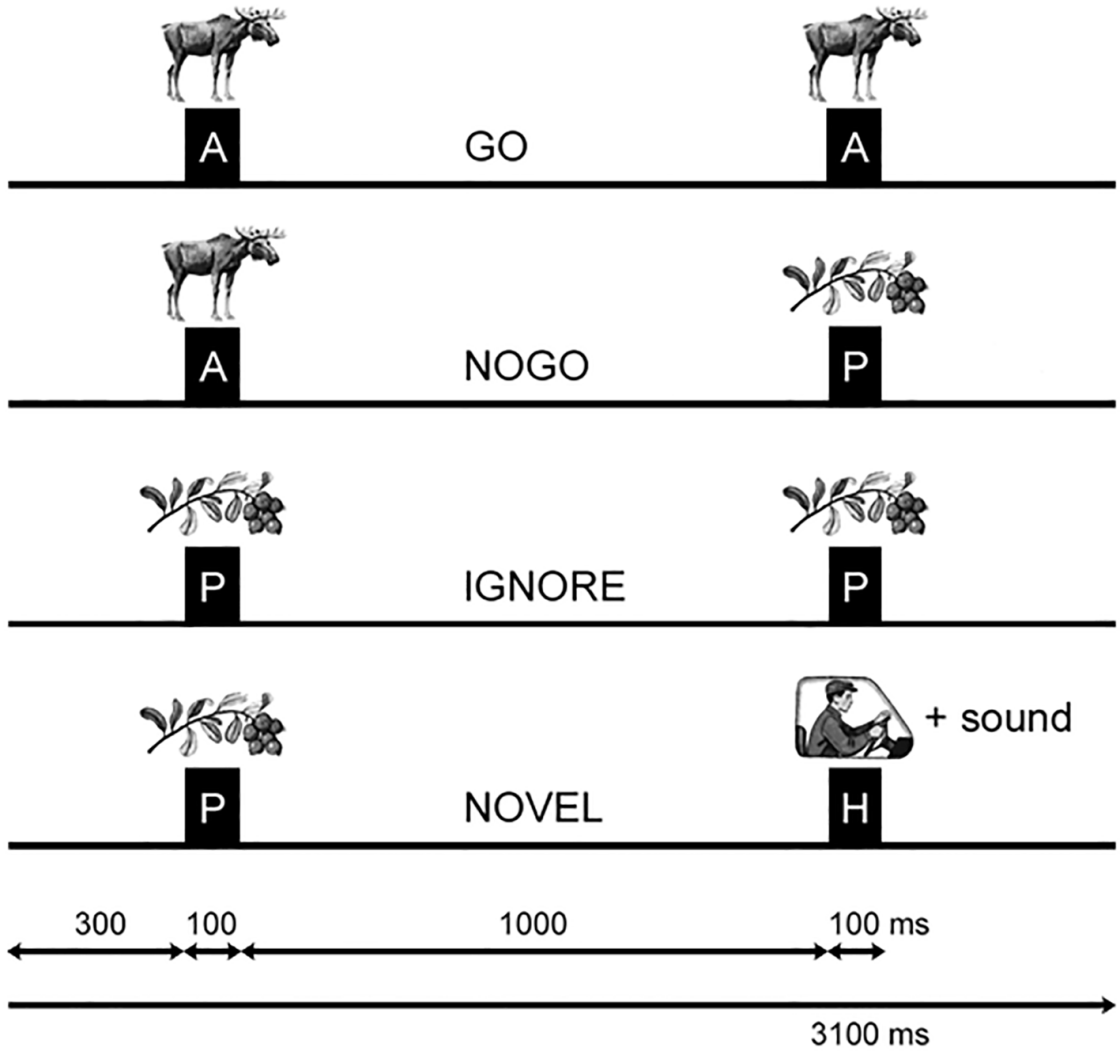
Visual continuous performance task (VCP1)/ Go/NoGo task. Schematic illustration of the two-stimulus GO/NOGO task showing the timing and sequence for four different trial types. A (animals), P (plants), and H (humans) represent the categories of visual stimuli. GO trials prompt the participant to press abutton, while NOGO trials involve inhibiting a planned response. In IGNORE trials, stimulus pairs start with aplant image and require no response preparation. NOVEL trials present anew sound along with the second human image, and no action is required. The timing intervals are displayed at the bottom for reference. Taken with permission from Mueller et al. (41).

The visual stimuli consisted of fifteen images from three categories: animals, plants, and humans (see **Figure 1**). The task included 400 trials of the Visual Continuous Performance Task (VCPT), where each trial presented a pair of stimuli (S1–S2). If the cue (S1) was an animal, S2 could either be an animal (Go trial) or a plant (No-Go trial). If the cue was a plant, participants were instructed not to respond (ignore trial).

Each stimulus (S1 and S2) was displayed for 100 ms, with a 1,100 ms interval between their onsets and a 3,000 ms gap between trials. The task included an equal number of Go and No-Go trials (200 each). Before starting the main task, clients practiced with 20–30 trials to familiarize themselves with the procedure. Rest periods were provided after every 200 trials.

Stimuli were presented on a 17-inch CRT screen positioned approximately 1.5 meters in front of the clients, covering approximately 3.8° of the visual field. Psytask software (developed by one of the authors, PVA) was used for stimulus presentation and response recording. Response times were considered correct if they occurred within a 100–1000 ms window after the target stimulus. Commission errors (responding during No-Go trials) and omission errors (failing to respond to Go trials) were calculated for each participant.

#### EEG acquisition and processing

To ensure methodological consistency, both the treatment and control groups were assessed using the same EEG hardware, software, and data acquisition procedures. EEG was recorded from nineteen scalp electrodes placed according to the 10–20 system (Fp1, Fp2, F7, F3, Fz, F4, F8, T3, T4, T5, T6, C3, Cz, C4, P3, Pz, P4, O1, O2) using a Mitsar-201 EEG system. Electrode impedances were routinely checked and maintained below 10 kΩ, in accordance with the manufacturer's recommendations for optimal signal quality (48). Signals were bandpass filtered between 0.5 and 50 Hz and sampled at 250 Hz. Ag/AgCl electrodes were applied using MCS caps with electro-gel for skin contact. Data were referenced to linked ears. The EEG data were later processed offline using WinEEG software. Artefacts caused by eyeblinks were corrected using independent component analysis (ICA), and any remaining epochs with excessive amplitude or frequency activity were excluded. The exclusion thresholds were set at 100 µV for non-filtered EEG, 50 µV for slow waves (0–1 Hz), and 35 µV for fast waves (20–35 Hz). ERP analysis was performed using the same software. Epochs were time-locked to the second stimulus (GO or NOGO) onset and extracted from –200 ms pre-stimulus to +800 ms post-stimulus, resulting in a total window size of 1000 ms. Baseline correction for the second stimulus was not applied.”

### Statistical analysis

Descriptive statistics were calculated to describe sociodemographic (i.e. age, sex) and clinical data (i.e. use of psychotropic medication).

#### Symptom analysis

To evaluate the impact of neurofeedback on PTSD symptoms, descriptive statistics, including median and interquartile range (IQR), were used to summarise pre- and post-treatment HTQ scores across six symptom dimensions: Total PTSD, Anxious Arousal, Numbing, Avoidance, Intrusions, and Dysphoric Arousal. Effect sizes were calculated to quantify the magnitude of change, with Cohen’s d and Cliff’s Delta applied to each dimension. Statistical significance of treatment effects was assessed using paired tests, and results were interpreted in terms of both statistical significance and effect magnitude.

To account for multiple comparisons (for Total PTSD, Anxious Arousal, Numbing, Avoidance, Intrusions, and Dysphoric Arousal), p-values were adjusted using the Bonferroni correction method. The significance threshold (α) was modified by dividing the original alpha level (α=0.05) by the number of comparisons (m=6), resulting in a corrected threshold of α′=0.0083. A clinically significant change was defined as a 0.5-point decrease on the HTQ, based on effect sizes reported in studies of neurofeedback, psychotherapy and pharmacological interventions for PTSD (42, 49, 50). Pre-post differences in HTQ scores of 0.4 or lower were classified as clinically non-significant.

Responders to treatment (defined as a ≥0.5-point decrease on the HTQ post treatment) were compared with non- or minimal responders on baseline characteristics of age, sex, residential status (temporary or permanent), years of education, living conditions (living alone or with other family members), use of interpreters, use of psychotropic medication and number of medications, number of trauma counselling sessions prior to NFT, number of NFT sessions and duration of NFT in months, presence of early childhood trauma and exposure to torture, and the baseline score on HTQ to understand factors related to treatment response. Differences between groups were assessed using a Wilcox rank sum test for continuous variables and Fisher’s exact test for categorical variables (i.e. sex and residential status).

In addition to these group comparisons, we conducted two complementary analyses to model the relationship between baseline symptom severity and treatment-related change. First, we performed a linear regression with change in HTQ scores (post–pre) as the dependent variable and baseline HTQ scores as the predictor. Second, to address limitations of difference score analyses, we implemented a Bayesian multilevel model with random intercepts and slopes. Time was modelled as a binary variable (pre/post), allowing us to estimate individual trajectories over two time points. This approach accounted for individual variability in symptom trajectories over time and allowed us to estimate whether baseline severity was associated with greater symptom reduction, while incorporating uncertainty through credible intervals. A sensitivity analysis was conducted *post hoc* to assess whether medication status confounded the association between treatment response and symptom change. A linear model was fitted using HTQ change scores as the dependent variable, with responder status and medication status as predictors.

#### ERP quantification and analysis

ERP signals were computed for both Go and No-Go conditions. The clients who had less than 30% of trials for each trial category for averaging were excluded from the analysis, to ensure reliable ERP waveforms. ERP NoGo-Go difference waves as indexes of cognitive control (51) were compared between the groups (Responders, Non-responders, Pre-, Post-, and PTSD vs Healthy/Normative Controls) using a cluster-based permutation test.

The cluster-based permutation test addressed the issue of multiple comparisons by organising the data into clusters based on temporal and spatial proximity. This procedure was based on the methodology implemented in the FieldTrip MATLAB toolbox for M/EEG analysis (accessible at http://fieldtrip.fcdonders.nl/, accessed 24/01/2023; 52, 53), but incorporated the following modifications: (1) the Wilcoxon signed-rank nonparametric test was employed to compare ERP waveforms across conditions, replacing the dependent sample t-tests used in the FieldTrip toolbox; (2) for cluster-level statistics, a normal approximation of the Wilcoxon signed-rank test and the sum of z-scores within a cluster were utilised instead of the sum of t-values. Nonparametric methods were selected for their ability to handle outliers effectively. Clusters with p-values below 0.05 were considered statistically significant. Analyses focussed on midline electrodes (Fz, Cz) within a 200 to 600 ms time window post-stimulus, consistent with standard ERP protocols.

#### Behavioural data quantification and analysis

Behavioural data, including omission and commission errors, response time, and response time variability, were summarised using medians and interquartile ranges (IQRs). Differences between the PTSD and Healthy Control (HC) groups, as well as between Responders and Non-Responders at baseline, were assessed using the Mann-Whitney U test, with effect sizes calculated using Cohen’s d and Cliff’s Delta.

Pre- to post-treatment changes within groups were evaluated using the Wilcoxon Signed-Rank Test. Multiple comparisons were controlled using both Bonferroni correction (adjusted α = 0.00625) and the False Discovery Rate (FDR) correction, as the behavioural measures are likely correlated and the analysis was exploratory in nature. Effect sizes were reported alongside significance levels, and non-significant trends were noted for exploratory interpretation.

#### Correlational analysis

To investigate the relationship between changes in Event-Related Potentials (ERP) and changes in scores on individual Harvard Trauma Questionnaire (HTQ) items, a Partial Least Squares Correlation (PLSC) analysis was conducted. This multivariate statistical approach explored associations between changes in the amplitude of the P3 wave and changes in individual HTQ item scores following neurofeedback treatment. PLSC enables the simultaneous analysis of multiple dependent variables (HTQ item scores) and independent variables (P3 ERP waveform), identifying combinations of variables that maximise shared variance.

ERP data, with a specific focus on the P3d wave recorded at Cz, a central electrode placement, was collected before and after neurofeedback treatment. HTQ item scores were calculated as difference scores to reflect therapeutic changes. The analysis projected the data from 47 participants into a latent space optimised to represent the relationship between ERP changes and HTQ outcomes. Statistical significance was assessed using a p-value threshold of <0.05, validating that the observed associations were unlikely to be due to chance.

PLSC analyses were conducted using R (54) with the support of the exposition package (55).

## Results

### Participant characteristics

#### Description of the sample

**Figure 2:**
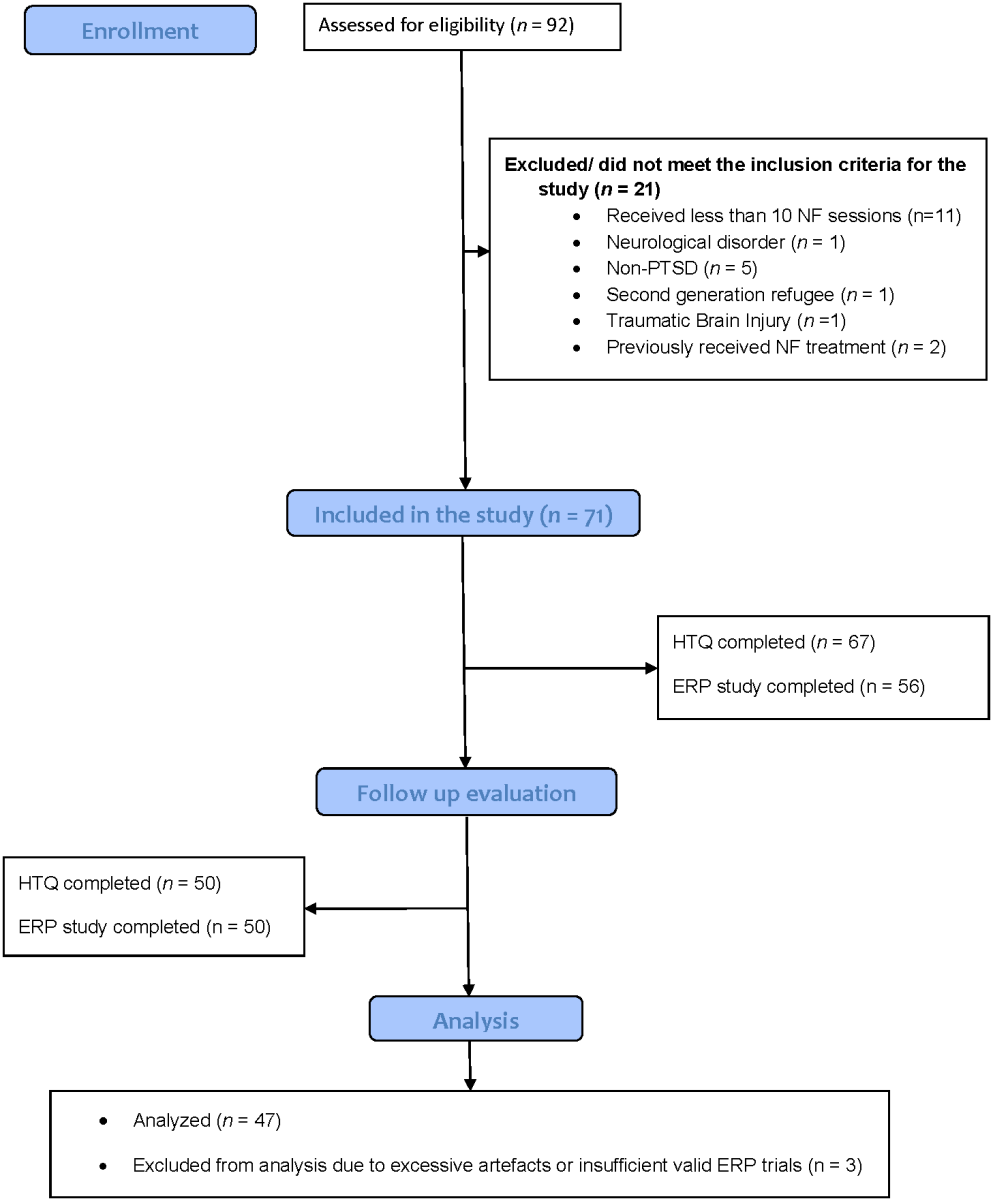
CONSORT flowch art of participants.

Of the 92 individuals assessed for eligibility, 21 were excluded for not meeting the study’s inclusion criteria (**Figure 2**). Reasons for exclusion included receiving fewer than 10 neurofeedback (NF) sessions (n = 11), presence of a neurological disorder (n = 1), absence of a PTSD diagnosis (n = 5), second-generation refugee status (n = 1), history of traumatic brain injury (n = 1), and prior exposure to NF treatment (n = 2). This resulted in a total of 71 participants being included in the study. At follow-up, 67 participants completed the Harvard Trauma Questionnaire (HTQ), and 56 completed the ERP protocol. Of these, 50 participants completed both the HTQ and ERP assessments. Three participants were excluded from the final ERP analysis due to excessive artefacts or insufficient valid ERP trials (i.e., fewer than 30% per condition). The final sample therefore consisted of 47 participants with complete and usable ERP and symptom data.

#### Socio-demographic characteristics

Demographic and clinical characteristics of the full sample are presented in **Table 2**. Most clients (85%) had permanent residency in Australia, while 15% were either asylum seekers or temporary visa holders. Approximately 32% of clients resided alone. The largest group of clients originated from Iraq (42%), followed by those from Iran (14%) and Syria (10%). In terms of education, 28% had completed primary education, 36% had secondary education, or held a diploma, and 32% possessed a bachelor's degree or higher, with 2% having no formal education and another 2% not reporting their educational background (**Supplementary Table 1**). Additionally, a considerable number of clients (79%) required the support of an on-site interpreter during both the assessment and treatment stages.

#### Past traumatic experiences

The types of traumas experienced by clients included highly distressing events. A substantial 34% reported their first trauma occurring before the age of six. Torture was identified by 40% of clients, while an overwhelming 89% had witnessed atrocities and the violent death of a family member. Additionally, 98% experienced other refugee-related traumas, including dangerous flight, targeted harassment, forced separation, communal violence, deprivation, and destruction of their homes.

#### Diagnostic and clinical characteristics

All clients in this study met DSM-5 criteria for PTSD (American Psychiatric Association [56). A total of thirty-one clients (66%) were prescribed psychotropic medications prior to their NFT treatment (see **Table 2**). Seven of these 31 clients ceased their psychotropic medication by the end of the NFT program. The prescription of medications was managed independently by each client's treating physician. The most used medications among clients were Selective Serotonin Reuptake Inhibitors (SSRIs) (n=12), analgesics (including opioids, n=9), and Tricyclic Antidepressants (n=9). In addition, 38% had a suspected head injury, mostly related to torture, although none have reported confirmed severe traumatic brain injury that required hospitalisation.

### Treatment characteristics

NFT provided to study clients involved a median of 26 sessions (IQR=20.5-38.5), and a median treatment duration of 7 months (IQR=5.5–12 months).

The distribution of neurofeedback placements showed that the temporal region accounted for the highest number of sessions, totalling 630 out of 1,170 (54%). This region primarily utilised protocols such as T4-P4 and T3-T4. Central and parietal placements followed, with 327 sessions (28%) using protocols like C3-C4 and P3-P4. Frontal placements comprised 111 sessions (9%), employing protocols including Fz-Pz and Fz-Cz. Other placements, including temporo-central and occipital regions, were used sporadically, accounting for the remaining 8%. For a detailed breakdown of the protocols, refer to **Supplementary Table 2**.

### Electrophysiological and behavioural characteristics of the sample

#### ERP indexes of PTSD

**Figure 3:**
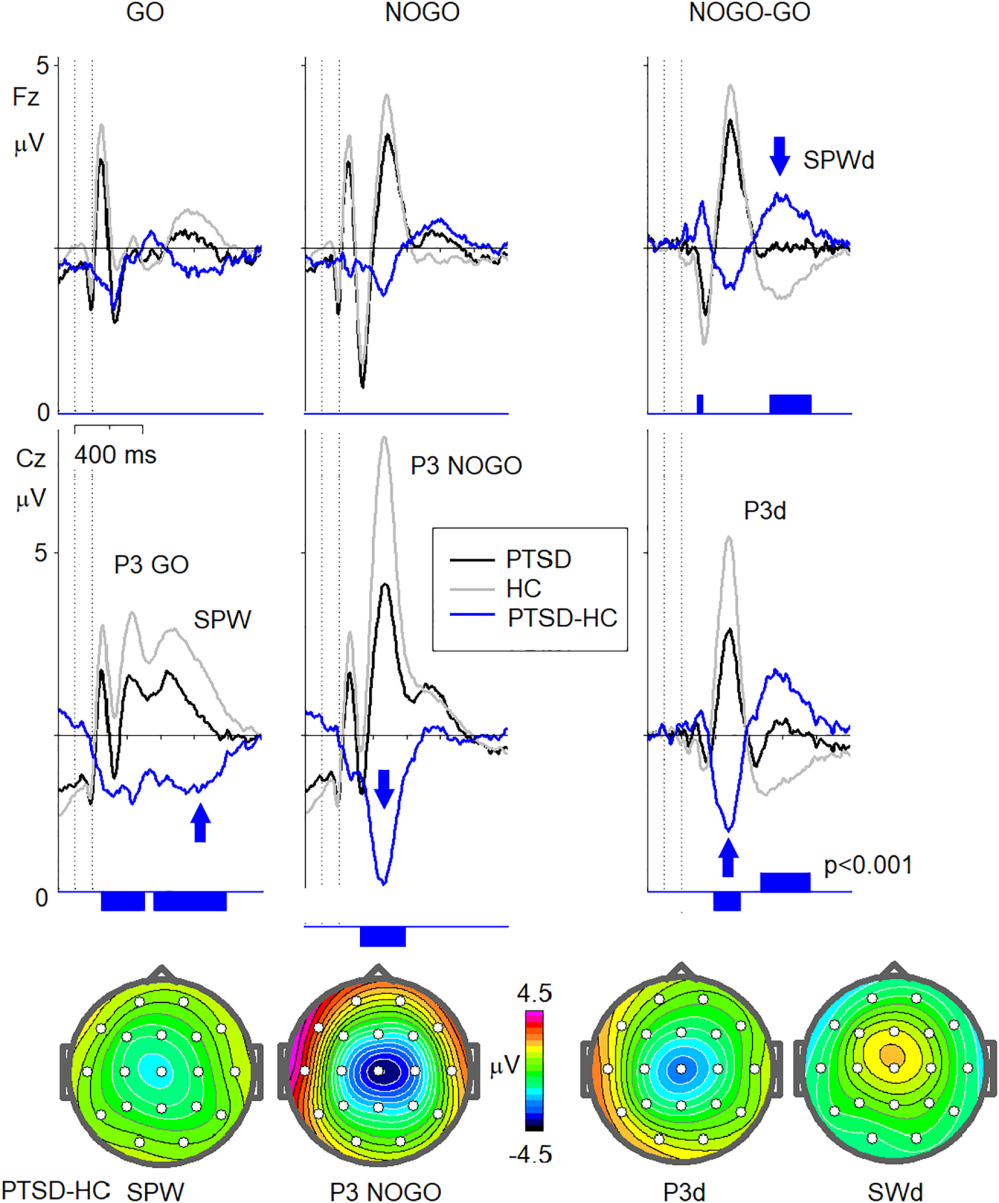
ERP indexes of brain dysfunction in PTSD. Grand averaged ERPs for Fz and Cz electrodes in response to GO (left column), NOGO (middle column) stimuli, as well as NOGO-GO ERP waves (right column) for clients with PTSD (N=47) (PTSD, black lines) and for the healthy controls (n=107) of the corresponding age (HC, grey lines). The canonical ERP waveforms in response to GO stimuli (P3 GO, SPW), NOGO stimuli (P3 NOGO), as well NOGO-GO difference waveforms (P3d, SPWd) are marked. The differences between the clients with PTSD and healthy controls (PTSD-HC) with the vertical bars indicating the statistically significant level (p<0.001) are presented in blue colour. Maps of the PTSD-HC differences at latencies marked by blue arrows and corresponded to SPW, P3 NOGO, P3d, and SPWd.

ERPs in the cued Go/NoGo task in response to Go, NoGo stimuli, as well as NoGo-Go ERP waves for all clients with PTSD (n=47) and for the healthy controls of the corresponding age are presented in **Figure 3**. The grand-averaged ERP waveforms from the Go/NoGo task revealed significant differences at the baseline between clients with PTSD and healthy controls (HC). Compared to HC, the PTSD group exhibited reduced amplitudes of the Slow Positive Wave (SPW) and P3 components across Go (P3 Go) and NoGo (P3 NoGo) conditions. The difference waveforms (NoGo-Go) showed the diminished amplitudes for P3d and SPWd components in PTSD group compared to HC. Statistical analyses indicated significant group differences (p < 0.001) during key time intervals, as illustrated by blue bars on the ERP waveforms. Scalp topographies of the PTSD-HC difference waveforms revealed that these reductions were most pronounced over the frontal and central regions (**Figure 2**).

#### Indices of impaired behaviour in PTSD

Compared to healthy controls, the PTSD group demonstrated significant impairments in behaviour on the Visual Continuous Performance Task (VCPT). Significant group differences were observed for omission errors and response time variability (**Table 3**). The PTSD group showed higher omission errors (Median = 12.0, IQR = 3.0–23.0) compared to Healthy Controls (HC; Median = 1.0, IQR = 0.0–2.0) with a large effect size (Cohen's *d* = 1.24, 95% CI = 0.86–1.61; Cliff’s Delta = 0.78; p < 0.001, corrected *p* < 0.001). Similarly, response time variability was greater in the PTSD group (Median = 135.0, IQR = 102.5–149.5) than in HC (Median = 74.0, IQR = 58.0–95.0), with a large effect size (Cohen's *d* = 1.39, 95% CI = 1.01–1.77; Cliff’s Delta = 0.67; p < 0.001, corrected *p* < 0.001). Differences in commission errors (PTSD: Median = 0.0, IQR = 0.0–2.0; HC: 0.0, IQR = 0.0–1.0) and response time (PTSD: Median = 427.0, IQR = 346.0–488.25; HC: 405.0, IQR = 349.0–465.5) were small (Cohen's *d* ≤ 0.57) and not significant after correction (corrected *p* > 0.01).

**Table 3:** VCPT at the baseline (n=46) vs healthy controls (HC) (n=107).

Measure	Baseline PTSD (Median, IQR)	HC (Median, IQR)	Cohen’s d (95% CI)	Cliff’s delta	P value (Mann-Whitney U test)	Bonferroni corrected p-value^ (p<0.01)	Effect size
Omission Errors	12.0 (3.0 – 23.0)	1.0 (0.0 – 2.0)	1.24 (0.86 – 1.61)	0.78	p < 0.001	Significant	Large
Commission Errors	0.0 (0.0-2.0)	0.0 (0.0 – 1.0)	0.57 (0.21 – 0.92)	0.18	p=0.03	Non-significant	Small
Response Time	427 (346.0 – 488.25)	405.0 (349.0 – 465.5)	0.26 (-0.09 – 0.61)	0.10	p = 0.34	Non-significant	Small
Response Time Variability	135.0 (102.5 – 149.5)	74.0 (58.0 – 95.0)	1.39 (1.01 – 1.77)	0.67	p < 0.001	Significant	Large

VCPT, Visual – Continuous Performance Task.

#### Demographic and clinical characteristics of responders and non-responders

Responders and non-responders (responders defined as those achieving a clinically significant reduction in PTSD symptoms; ≥0.5-point decrease on the HTQ) did not differ significantly in terms of gender, residential status, years of education, interpreter use, living arrangements, or trauma history (early trauma, torture exposure). These findings are summarised in **Table 1**. While responders tended to be younger (median = 45.5 years) than non-responders (median = 50.0 years), this difference was not statistically significant (U=246.0; p=0.543).

However, responders were more likely to use psychotropic medications overall, with 82% of responders (18 of 22) reporting psychotropic medication use compared to 52% of non-responders (13 of 25). This difference approached statistical significance (χ² = 3.40, p=0.065). The number of medications used did not significantly differ between responders (median = 1.0) and non-responders (median = 2.0; U=86). To assess whether psychotropic medication influenced treatment response, we conducted a *post hoc* sensitivity analysis using a linear model. Responder status remained a strong and statistically significant predictor of HTQ symptom reduction (F(1, 44) = 70.73, p <.001), while medication status had no significant effect (F(1, 44) = 0.32, p = .575). These results suggest that the symptom improvements observed among responders were not attributable to medication use.

Additionally, responders reported significantly higher baseline PTSD severity score on HTQ (median = 3.2, IQR = 0.4) compared to non-responders (median = 2.6, IQR = 0.6), with a highly significant difference (U=474.5 p < 0.001). These findings suggest that greater initial symptom severity may influence treatment responsiveness, while other demographic and trauma-related variables showed no meaningful differences (**Table 1**). Although responders were more likely to be medicated, a *post hoc* sensitivity analysis indicated that medication status did not significantly predict symptom change, suggesting the observed differences were not attributable to psychotropic medication use. In the cued Go/NoGo task, baseline group differences between Responders and Non-Responders were examined across omission and commission errors, response time, and response time variability. At baseline, differences in VCPT performance between responders and non-responders were not statistically significant across all measures. Responders had a slightly higher median omission rate (14.0, IQR 6.0–27.0) compared to non-responders (8.0, IQR 3.0–21.0), with a negligible effect size (Cliff's Delta = 0.19). Commission errors were low for both groups, with a small effect size (Cliff's Delta = 0.15). Response times were faster among responders (median 376.0 ms, IQR 342.0–447.0) than non-responders (median 451.0 ms, IQR 346.0–580.0), showing a medium effect size (Cliff's Delta = -0.28). Variability in response time (Response Time SD) was similar between groups (responders: median 130.0, IQR 104.0–150.0; non-responders: median 139.0, IQR 88.0–148.0), with a negligible effect size (Cliff's Delta = -0.04). None of the differences reached significance after Bonferroni correction. Full results are provided in **Table 4**.

**Table 4:** Responders vs non-responders VCPT at baseline.

Measure	Baseline responders (Median, IQR)	Non-responders (Median, IQR)	Cohen’s d (95% CI) ^a^	Cliff’s delta ^b^	P value	Bonferroni Corrected p-value	Effect size
Omission	14.0 (6.0-27.0)	8.0 (3.0-21.0)	0.20 (-0.39, 0.78)	0.19	0.30	Not Significant	Negligible to Small
Commission	0.0 (0.0-2.0)	0.0 (0.0-1.0)	0.39 (-0.20, 0.98)	0.15	0.35	Not Significant	Small
Response Time	376.0 (342.0-447.0)	451.0 (346.0-580.0)	-0.51 (-1.10, 0.08)	-0.28	0.10	Not Significant	Small to Medium
Response Time SD	130.0 (104.0-150.0)	139.0 (88.0-148.0)	0.01 (-0.57, 0.59)	-0.04	0.84	Not Significant	Negligible

VCPT, Visual Continuous Performance Taks; ^a^Cohen’s d, values of 0.0–0.19 indicate negligible effects, 0.20–0.49 small effects, 0.50–0.79 medium effects, and ≥0.80 large effects; ^b^Cliff’s Delta, values <0.147 are negligible, 0.147–0.33 small, 0.33–0.474 medium, and ≥0.474 large.

#### ERP indexes for responders vs non-responders

**Figure 4:**
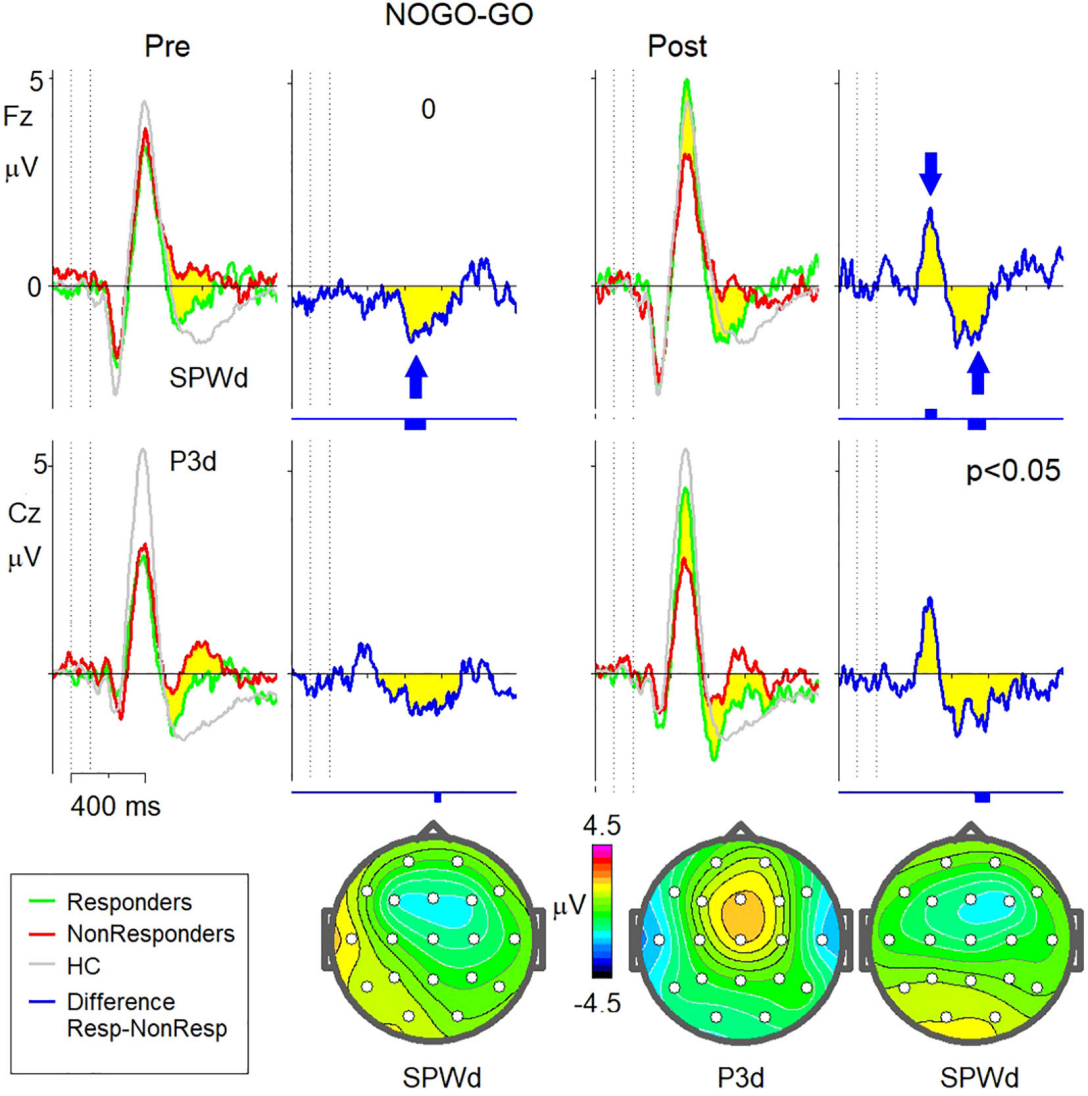
NOGO-GO waveforms for Responders vs Non-Responders before and after sessions of neurofeedback. Left: Grand-averaged ERP NOGO-GO wavefonns at baseline (prior to neurofeedback sessions) are shown for Responders (green lines), Non-Responders (red lines), and Healthy Controls (HC; grey lines). The difference wave (Responders minus Non-Responders) is depicted in blue. Vertical bars below the wavefonn indicate time intervals of statistical significance (p < 0.05). The arrow marks a statistically significant difference at the SPWd peak The topographical map below illustrates the scalp distribution of the Responders vs. Non-Responders difference at the SPWd peak latency. Right: Grand-averaged ERP NOGO-GO wavefonns post-neurofeedback sessions are shown for Responders (green lines),Non-Responders (red lines), and Healthy Controls (HC; grey lines). The difference wave (Responders minus Non-Re sponders) is depicted in blue. Vertical bars below the waveform indicate regions of statistical significance (p < 0.05). Arrows mark statistically significant differences at the P3d and SPWd peaks. The topographical map below illustrates the scalp distribution of the Responders vs. Non-Responders differences at the P3d and SPWd peak latencies.

At baseline, both canonical waveforms P3d and SPWd—exhibited statistically significant differences between the control and PTSD groups (responders and non-responders), with the PTSD groups showing smaller amplitudes (**Figure 4**). There were no significant differences in P3d amplitude between the responders and non-responders at baseline. However, the SPWd waveform demonstrated significant differences between responders and non-responders, with responders showing smaller deviations from the healthy control group at baseline.

### Effectiveness of neurofeedback therapy: pre-post intervention analysis

#### Symptoms of PTSD (HTQ), total and subscales pre-post NFT

The analyses showed significant reductions in PTSD symptoms across all measures following the intervention (**Table 5**). Large effect sizes were observed for HTQ Total score (d=1.28, p<0.001) and Dysphoric Arousal (d=0.99, p<0.001). Medium to large effects were found for Anxious Arousal (d=0.82, p=0.0024) and Avoidance (d=0.76, p=0.0042). Medium effects were seen for Numbing (d=0.67, p=0.020) and Intrusions (d=0.60, p=0.0455). All p-values are Bonferroni corrected.

**Table 5:** PTSD scores on HTQ at the baseline and post NFT.

Dimension	Baseline (Median, IQR)	Post-NFT (Median, IQR)	Cohen’s d (95% CI)	Cliff’s delta (95% CI)	Bonferroni corrected p-value^	Effect size interpretation
HTQ Total	2.90 (2.55–3.20)	2.20 (1.90–2.65)	1.28 (0.77, 1.79)	0.59 (0.48, 0.70)	p < 0.001 *	Large
Anxious Arousal	3.00 (3.00–3.50)	2.50 (2.00–3.00)	0.82 (0.34, 1.31)	0.42 (0.31, 0.53)	p = 0.0024 *	Medium to Large
Avoidance	3.00 (2.00–3.50)	2.00 (1.00–3.00)	0.76 (0.27, 1.24)	0.41 (0.30, 0.52)	p = 0.0042 *	Medium to Large
Numbing	2.50 (2.00–3.00)	2.00 (1.50–2.50)	0.67 (0.19, 1.14)	0.35 (0.24, 0.46)	p = 0.020	Medium
Intrusions	3.00 (2.50–3.50)	2.62 (2.19–2.88)	0.60 (0.13, 1.08)	0.32 (0.21, 0.43)	p = 0.0455	Medium
Dysphoric Arousal	3.30 (2.70–3.70)	2.70 (2.00–2.85)	0.99 (0.50, 1.48)	0.59 (0.48, 0.70)	p < 0.001 *	Large

Posttraumatic stress disorder (PTSD) was measured using the Harvard Trauma Questionnaire (HTQ). Cohen’s d is a standardised effect size indicator interpreted as small (d = 0.2), medium (d = 0.5), and large (d = 0.8). Cliff’s Delta is a non-parametric effect size indicator interpreted as negligible (<0.15), small (0.15–0.33), medium (0.33–0.47), and large (>0.47). CI refers to the 95% confidence interval for effect sizes. Adjusted p-values (^) account for multiple comparisons using the Bonferroni correction. The significance level for comparisons marked with an asterisk (*) was set at p < 0.05.

**Figure 5:**
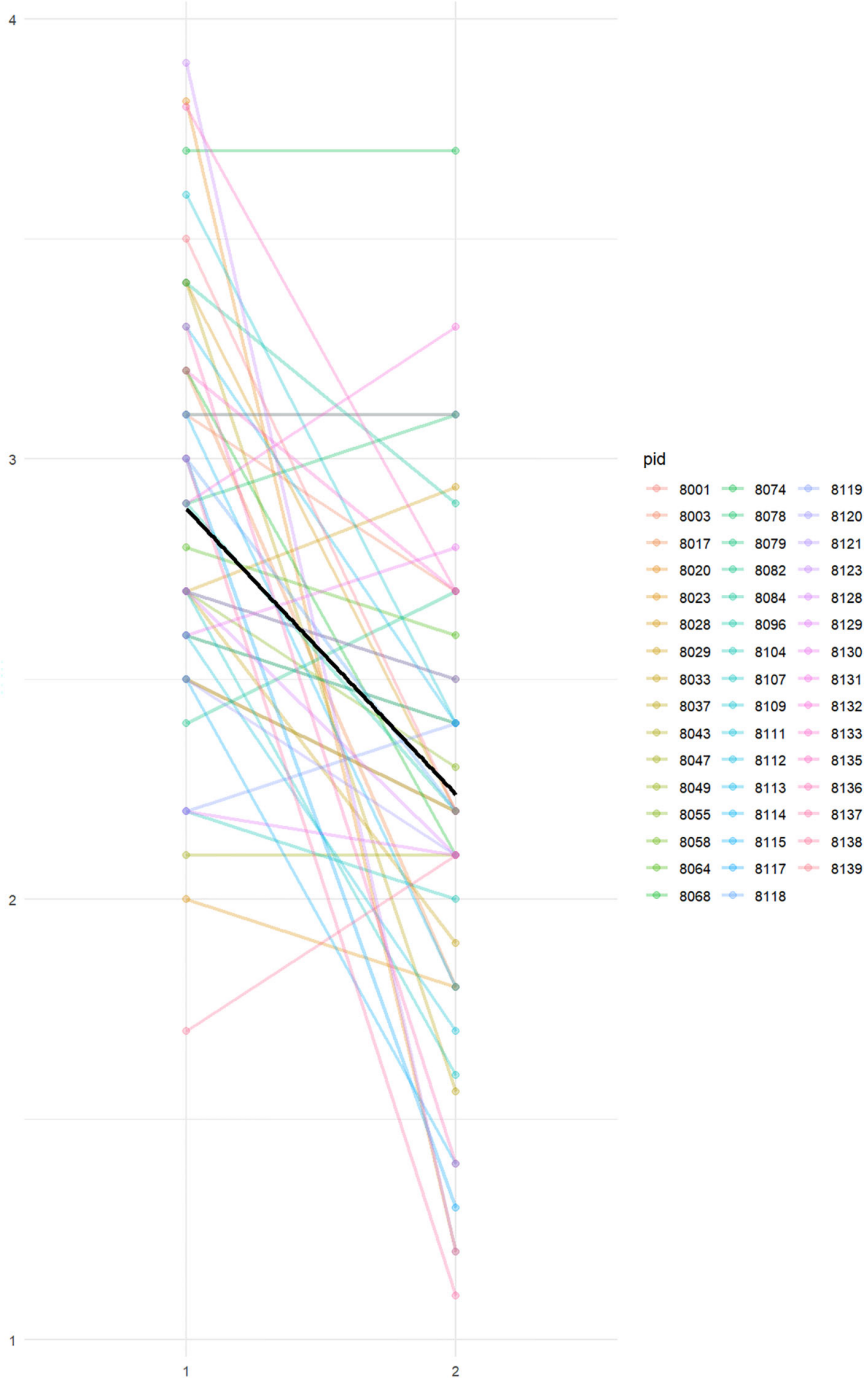
Individual trajectories of PTSD symptom severity (HTQ scores) pre- and post-intervention. Each line represents a single participant. A general trend toward symptom reduction is visible across the sample (time 1 to time 2), although the degree of change varies, highlighting the heterogeneity of treatment response.

**Figure 5** displays individual trajectories of PTSD symptom severity (HTQ scores) from pre- to post-intervention. Each line represents a participant’s change over time. The plot illustrates a general trend of symptom reduction across the sample, particularly among those with more severe baseline symptoms, though variability in treatment response is clearly visible.

#### Changes in behavioural performance for responders and non-responders on Go/NoGo task pre-post NFT

Responders showed reductions in omission errors and response time variability following the intervention, while commission errors increased slightly in terms of the median. Specifically, omission errors decreased from a median of 14.0 (IQR: 6.5–31.5) pre-intervention to 8.0 (IQR: 1.25–17.0) post-intervention (p=0.02), and response time variability (RTV) improved from 127 ms (IQR: 102–148) to 102 ms (IQR: 56–131) (p=0.02). In contrast, commission errors increased slightly from 0.0 (IQR: 0–2) to 0.5 (IQR: 0–0.75) (p=0.01). Although several changes reached significance at the uncorrected level, none remained significant under Bonferroni correction. FDR correction was also applied to account for the exploratory nature and intercorrelation among variables, and some trends (e.g., in omission errors and RTV among responders) remained below the FDR threshold of 0.10.

Non-responders also demonstrated some improvements, with omission errors decreasing from a median of 8.0 (IQR: 3–21) to 5.0 (IQR: 2–20) (p=0.17) and RTV showing a minor reduction from 139 ms (IQR: 88–148) to 102 ms (IQR: 91–163) (p=0.37). Response time improved from 451 ms (IQR: 346–580) to 409 ms (IQR: 328–508) (p=0.04). While no changes reached statistical significance under Bonferroni correction, some trends remained observable at the FDR-corrected level, indicating potential improvement deserving further investigation. More details are presented in **Table 6**.

**Table 6:** Pre-post NFT differences on VCPT for responders and non-responders.

Group	Error type	Median (IQR) Pre	Median/IQR Post	W, (W-, W+) Wilcoxon test statistic	p-value	Bonferroni corrected p-value (α = .00625)	FDR-corrected p-value
Responders	Omission	14.0 (6.5-31.5)	8.0 (1.25-17.0)	42.5, (167.5, 42.5)	0.02*	0.16	0.05
Non-Responders	Omission	8.0 (3-21)	5.0 (2-20)	92.5, (183.5, 92.5)	0.17	1.36	0.23
Responders	Commission	0.0 (0-2)	0.5 (0-0.75)	5, (61, 5)	0.01*	0.08	0.05
Non-Responders	Commission	0.0 (0-1)	0.0 (0-2)	38.5, (39.5, 38.5)	1	8.0	1.0
Responders	Response Time	376 (340-441)	359.5 (325-431)	62, (169, 62)	0.06	0.48	0.1
Non-Responders	Response Time	451 (346-580)	409 (328-508)	79, (221, 79)	0.04*	0.32	0.08
Responders	Response Time Variability	127(102-148)	102 (56-131)	50, (181, 50)	0.02*	0.16	0.05
Non-Responders	Response Time Variability	139 (88-148)	102 (91-163)	118, (182, 118)	0.37	2.96	0.42

NFT, Neurofeedback Treatment; VCPT, Visual Continuous Performance Task. Within-group pre-post differences were analysed using the Wilcoxon signed-rank test. Uncorrected p-values are reported alongside Bonferroni-corrected p-values (adjusted α = 0.00625 for 8 comparisons) and False Discovery Rate (FDR)-corrected p-values (Benjamini-Hochberg procedure). Asterisks (*) denote statistical significance at the uncorrected level (p <.05). None of the effects remained significant after multiple comparison correction.

#### Changes in ERP indexes for responders vs non-responders pre-post NFT

After neurofeedback therapy, the P3d amplitude in responders normalised to levels comparable to healthy controls. In contrast, non-responders showed no change in P3d amplitude, leading to a statistically significant difference in this measure between the two groups following treatment. The SPWd waveform, however, remained unchanged post-treatment in both groups, maintaining the significant difference observed at baseline (See **Figure 4**).

#### The partial least squares correlation

**Figure 6:**
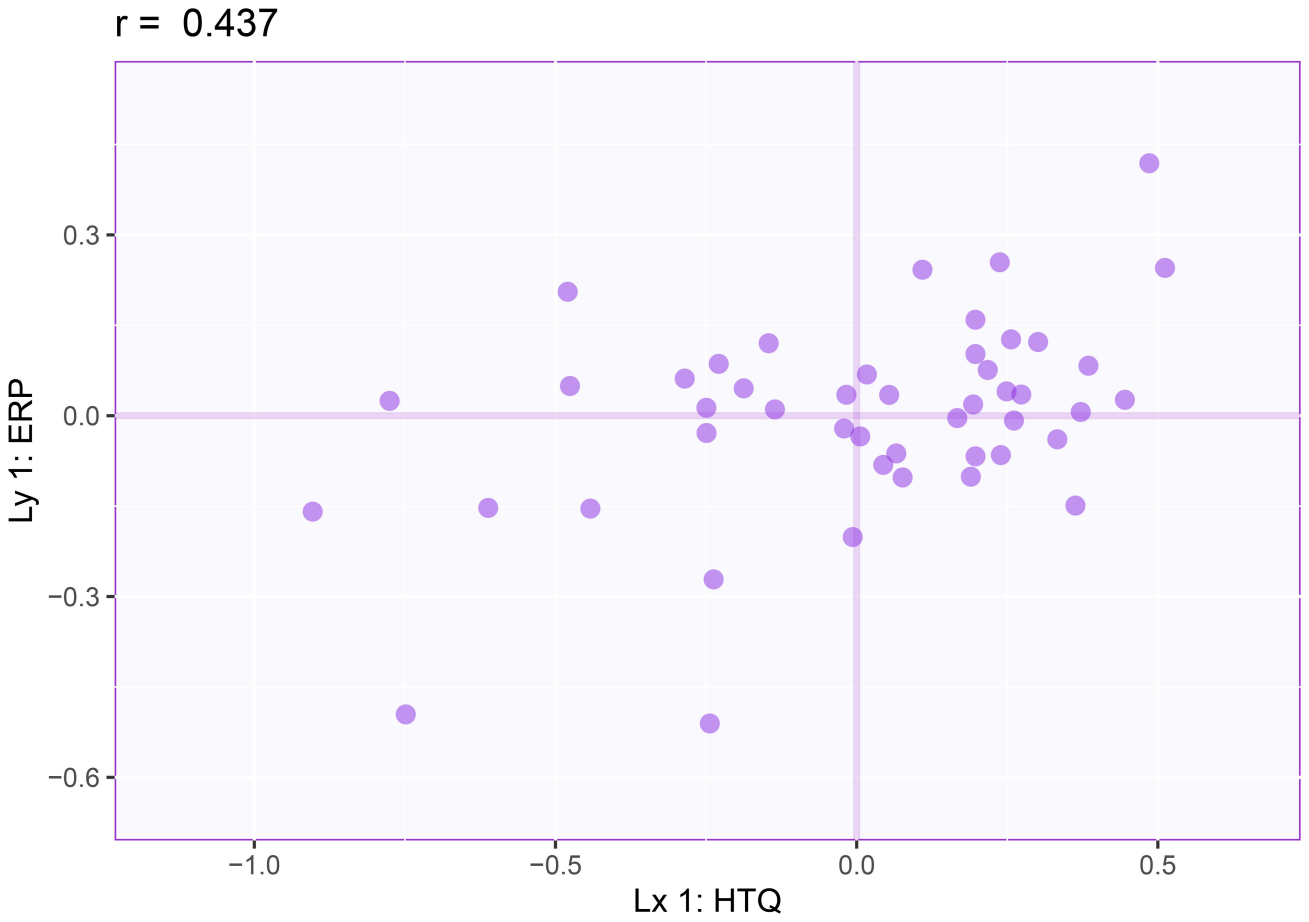
Partial least squares correlation results for the first latent variable dimension: P3d amplitude changes (Y) and HTQ changes (X). This scattetplot represents 1hepartial least squares correlation analysis examining the relationship between changes in P3d amplitude at Cz and changes in HTQ scores pre- and post-neurofeedback treatment. Each point corresponds to an individual client. The correlation coefficient (r = 0.437, p < 0.05)highlights a statistically significant association.

The partial least squares correlation (PLSC) analysis identified significant multivariate associations between changes in the amplitude of the P3 wave and changes in individual HTQ item scores following neurofeedback treatment (**Figure 6**). The PLSC model revealed a statistically significant first latent dimension (p=0.033), which accounted for 93.76% of the variance in the dataset, while the second dimension (p=0.814) explained only 6.24% and was not statistically significant.

As presented in **Table 7**, the contributions and bootstrap ratios indicated that the latent variable of the EEG data was primarily influenced by the late component recorded at Cz-Av_2 (t=-2.325). For the HTQ data, significant contributions were observed for items related to trauma symptoms, including avoiding activities (t=2.295), feeling as if something is happening (t=2.167), feeling jumpy (t=2.030), difficulty sleeping (t=2.009), and being on guard (t=2.044).

**Table 7:** Contributions and bootstrap ratios of EEG and HTQ variables to dimensions 1 and 2 in the PLSC model.

Set	Item	Dimension 1 (c)	Dimension 1 (t)	Dimension 2 (c)	Dimension 2 (t)
HTQ	1. “Recurrent thoughts or memories of the most hurtful or terrifying events”	0.085	1.998	0.011	-0.293
HTQ	2. “Feeling as though the event is happening again”	0.125	2.167*	0.019	-0.399
HTQ	3. “Recurrent nightmares”	0.002	0.422	0.084	1.097
HTQ	4. Feeling detached or withdrawn from people”	0.142	1.857	0.061	0.566
HTQ	5. “Unable to feel emotions”	0.005	0.451	0.139	-1.295
HTQ	6. “Feeling jumpy, easily startled”	0.133	2.03*	0.008	0.24
HTQ	7. Difficulty concentrating”	0.041	1.417	0.011	-0.235
HTQ	8. “Trouble sleeping”	0.093	2.009*	0.032	0.506
HTQ	9. “Feeling on guard”	0.076	2.044*	0.002	-0.097
HTQ	10. “Feeling irritable or having outbursts of anger”	0.07	1.651	0.047	-0.56
HTQ	11. “Avoiding activities that remind you of the traumatic or hurtful event”	0.188	2.295*	0.009	0.327
HTQ	12. “Inability to remember parts of the most hurtful or traumatic event”	0.005	-0.608	0.283	1.799
HTQ	13. “Less interest in daily activities”	0.011	0.969	0.134	1.139
HTQ	14. Feeling as if you don’t have a future”	0.021	1.604	0.011	-0.315
HTQ	15. “Avoiding thoughts or feelings associated with the traumatic or hurtful events”	0.002	-0.361	0.029	0.38
HTQ	16. “Sudden emotional or physical reaction when reminded of the most hurtful or traumatic events”	0.002	0.311	0.122	-0.841
EEG	Cz-Av_2	0.849	-2.325*	0.151	-0.835
EEG	Pz-Av_1	0.151	-1.335	0.849	1.754

EEG, electroencephalography. Contributions (c) indicate the importance of each variable in the latent dimensions, while bootstrap ratios (t) reflect statistical significance, with values beyond ±2 (*) considered significant (p<0.05). HTQ items represent trauma symptoms, and EEG variables reflect late component activity at specific electrode sites.

The statistical analysis confirmed the significance of these findings, with a Bonferroni corrected p-value of <0.05 indicating that the observed associations were unlikely to occur by chance.

## Discussion

This study demonstrates the potential benefits of NFT for refugees with chronic treatment-resistant PTSD, highlighting significant reductions in PTSD symptoms, alongside enhancements in P3d waveform reflecting improvement in cognitive control. These findings, combining clinical and neurophysiological outcomes, offer preliminary insight into NFT’s role in enhancing both psychological and cognitive functioning in a population with chronic, trauma-related challenges, aligning with our previous work (35, 36).

### Reduction in PTSD symptoms following neurofeedback

A particularly strong effect was observed for symptoms reflecting dysphoric arousal linked to functional impairments in daily functioning, including difficulty concentrating, anger/irritability, and sleep problems (p <.001, large effect size). This is noteworthy given evidence highlighting the persistent nature of sleep, anger, and concentration difficulties in individuals with PTSD (57). Sleep disturbances are well-documented contributors to both the onset and persistence of PTSD (58). Chronic sleep disruption may undermine the effectiveness of first-line PTSD treatments, emphasising the importance of targeted interventions for sleep as a critical component of PTSD recovery (59). Similarly, attention dysregulation is increasingly recognised as a core factor in mental health disorders, including PTSD (60). It has been identified as a significant predictor of changes in overall psychopathological status, highlighting its critical role in the trajectory of mental health outcomes (61). In PTSD, attentional difficulties extend beyond a simple bias toward threatening stimuli, disrupting broader cognitive functions and undermining daily life functioning. Despite their prevalence and impact, these deficits often remain unaddressed in conventional treatment approaches (62). Furthermore, impairments in attentional control not only perpetuate the persistence of PTSD symptoms but also diminish the efficacy of therapeutic interventions, underscoring the need for targeted strategies to enhance attention regulation as part of comprehensive PTSD care (63).

Anger/irritability was also reduced. For refugees with PTSD addressing anger is recognised as a crucial component in the recovery process, as unresolved anger exacerbates symptoms and interferes with treatment outcomes. Spiller et al. (64) and Le et al. (65) highlight the significant role of anger in PTSD severity, emphasising that comorbid anger is strongly associated with poorer outcomes. Le et al. further demonstrate that perceived uncontrollability and distress during torture increase the risk of anger-related symptoms, independent of post-migration stressors. These findings suggest that integrating anger- focussed assessments and targeted interventions into PTSD therapies could enhance emotional stability and resilience, particularly for refugees who face complex trauma and significant post-migration challenges. Neurofeedback, by facilitating self-regulation of brain activity, appeared to be effective in addressing dysregulated sleep, attention and affect, as it targets underlying neural patterns associated with hyperarousal and emotional reactivity (31).

Significant improvements were also noted in Anxious Arousal and Avoidance, demonstrating medium to large effect sizes. While positive trends were observed for Numbing and Intrusions (medium effect sizes), these did not reach the same level of statistical significance as other symptom clusters (p >0.001 after Bonferroni correction). This may indicate that these symptom clusters are more resistant to change with NFT alone, although the current study was underpowered to detect significant changes in these domains and future research with larger sample sizes may clarify these findings.

### Cognitive control dysfunction in PTSD: ERP and behavioural evidence

The results of the present study indicate that individuals with chronic treatment-resistant PTSD exhibit cognitive control deficits during a cued Go/NoGo task, evident in both electrophysiological and behavioural measures. Specifically, reduced P3d amplitude, compared to healthy controls, suggests impaired inhibitory control (13, 14, 66, 67). This aligns with Falconer et al.'s findings of reduced medial prefrontal cortex (mPFC) and dorsal anterior cingulate cortex (dACC) activation during a Go/NoGo task, suggesting that even neutral inhibition tasks may exceed the capacity of these regulatory cortical structures in PTSD. (68).

The dorsolateral (dlPFC) and ventromedial (vmPFC) regions within the prefrontal cortex (PFC) are particularly important for top-down regulation (10), enabling individuals to inhibit automatic responses to distressing stimuli and maintain goal-oriented behaviour (69, 70). Impairments in these regions among individuals with PTSD are shown to relate to restricted cognitive flexibility, heightened emotional reactivity, and increased efforts to modulate emotional responses to trauma-related triggers (10).

The anterior cingulate cortex (ACC), a crucial region for attention regulation and response inhibition, exhibits altered activity in PTSD, influenced by the emotional valence of the task that can result in either heightened ventral ACC activation, indicative of an increased, but strained effort to divert attention from trauma-related cues (71), or diminished dorsal ACC activation, suggesting reduced capacity to inhibit responses to even non-threatening stimuli. (68).

Reduced P3 amplitudes could also suggest diminished attentional capacity, making it difficult for individuals with PTSD to allocate attention to non-threatening, novel information, as previously reported (18). Additionally, diminished NoGo P3 amplitude has been linked to high interpersonal trauma load, reflecting depleted cognitive resources (72). This suggests that the severity of trauma exposure in this refugee sample may have contributed to the observed impairments in cognitive control.

In addition, reduced SPW amplitudes in the PTSD group may indicate impaired late-stage cognitive processing. While research on SPW in PTSD is limited, the observed reductions in our study align with findings in other cognitive domains. For example, slow wave activity, which encompasses similar late potentials, has been linked to working memory maintenance (22) and cognitive effort (20).

The behavioural performance further supports these electrophysiological impairments. The PTSD group at baseline exhibited more omission errors and greater response time variability on the VCPT task, reflecting difficulties with sustained attention and inconsistent cognitive performance. These deficits may arise from the same disrupted neural mechanisms underlying the reduced P3 amplitude, indicating that individuals with PTSD struggle to allocate and sustain attention over time (73). In contrast, the slight differences observed in commission errors and response time suggest that, although impulsive responding and basic processing speed appear relatively intact, the capacity to sustain cognitive stability during prolonged or demanding tasks is significantly impaired.

### Neurofeedback impact on cognitive control

We identified the differential effects of neurofeedback therapy (NFT) on cognitive control in chronic TR-PTSD between responders and non-responders. Following NFT, the P3d amplitude in responders normalised to levels comparable to healthy controls, suggesting enhanced neural processing associated with improved inhibitory control. In contrast, non-responders showed no change in P3d amplitude, leading to significant post-treatment differences between the two groups. The increase in P3 NoGo amplitudes observed in responders post-treatment align with prior research demonstrating that improvements in PTSD scores following CBT are associated with increased P3 amplitude (30). Similarly, mindfulness-based interventions have shown increased P3 amplitudes in response to target stimuli and reduced distractor power (74). At the same time, the absence of P3d amplitude changes among non-responders may also reflect underlying difficulties in modulating neurofeedback training (NFT) targets—a phenomenon sometimes referred to as the "Brain-Computer Interface (BCI) inefficiency effect" (75, 76). When participants are unable to engage effectively with the NFT process, they may resort to maladaptive cognitive or neural strategies, recruiting inappropriate brain networks that could impair task-relevant processing and inhibitory control (77). This may, in turn, contribute to poorer clinical and electrophysiological outcomes. Although ERPs are seldom used in refugee populations with PTSD, this study and our prior research (36) demonstrate that pre-post- treatment ERP measurements can capture shifts in cognitive control in refugees with chronic TR-PTSD following NFT.

The persistence of baseline differences in the SPWd waveform in both groups suggests that SPWd may reflect deeper neurophysiological processes less amenable to short-term intervention, or that it is a trait marker of chronic PTSD. While SPWd waveforms remained unchanged in both groups after treatment, responders showed smaller baseline SPWd deviations compared to non-responders and healthy controls, suggesting more compromised late-stage cognitive processing in the latter group (20, 78, 79). This larger deviation could be associated with non-responsiveness due to a decreased cognitive capacity, including motivated attentional processing (20) and working memory (79). Similar SPW waveform changes have been identified in PTSD using emotional paradigm, with one study (25) finding a reduced SPW (LPP) associated with decreased responsiveness to positive environment and heightened vulnerability to depressive symptoms. Incorporating SPWd assessments into initial evaluations could help identify individuals at risk of not responding to NFT, or those requiring longer treatment, enabling clinicians to consider adjunctive or alternative interventions early in the treatment process.

Neurocognitive testing using the VCPT provided valuable insights into cognitive function and treatment outcomes. Both groups demonstrated significant impairments in attention compared to healthy controls, as evidenced by elevated omission errors and greater variability in reaction time. While no behavioural differences between responders and non-responders reached significance after Bonferroni correction, several improvements—particularly among responders—remained evident under FDR correction, suggesting emerging trends that may have clinical relevance and should be explored in larger studies. Previous research has also reported no performance differences despite clear neural processing changes during response inhibition task (80, 81) suggesting that responders may engage inhibitory processes more efficiently (30) However, the failure to observe statistically significant behavioural changes after Bonferroni correction—despite clear trends and FDR-adjusted p-values approaching significance—may also reflect limitations in sample size and statistical power. These findings nonetheless support the potential clinical relevance of the observed behavioural improvements, particularly in responders.

In addition, clinical factors also played a role in differentiating responders from non-responders. Responders were more likely to use medications, suggesting either a synergistic interaction between pharmacological interventions and NFT or greater clinical engagement among clients in this group. However, our sensitivity analysis indicated that medication status did not significantly predict symptom change, and the observed treatment effects remained robust after accounting for medication use. Clients were prescribed a wide variety of medications by external providers, and this heterogeneity limits conclusions about specific pharmacological contributions. Controlled studies are needed to explore potential synergistic or augmenting effects of neurofeedback and medication.

Consistent with the findings of Kemna et al (82) and Sonne et al. (83), our analysis suggests that individuals with more severe baseline PTSD symptoms may derive greater benefit from neurofeedback. This pattern first emerged in the group-level comparison, where responders exhibited significantly higher baseline HTQ scores than non-responders. To address the limitations of interpreting raw change scores—which can be influenced by regression to the mean—we conducted a linear regression, revealing that baseline symptom severity explained a substantial proportion of the variance in treatment-related change. This relationship was further supported by a Bayesian multilevel model, which estimated a negative correlation between initial HTQ scores and symptom change, although the wide credible interval highlights uncertainty due to the modest sample size (see **Figure 5**).

These results suggest a potential trend: individuals with more severe clinical presentations may exhibit greater symptom reduction following neurofeedback. However, this observation is hypothesis-generating rather than confirmatory and should be interpreted with caution. For participants with milder symptoms, a floor effect may have constrained measurable improvement, potentially leading to their misclassification as non-responders. This limitation, also noted by Andrzejowski et al. (84), illustrates how outcome sensitivity can be compromised when initial symptom levels are low. Taken together, these findings underscore the importance of larger, controlled studies with more evenly distributed symptom severity to confirm whether baseline clinical status reliably moderates treatment response.

Demographic factors such as gender, residential status, years of education, living arrangements, early trauma, and exposure to torture showed no significant associations with treatment outcomes. These findings highlight the potential usefulness of individual neurophysiological profiles, over and above broader demographic or trauma-related variables in identifying treatment responsiveness.

For non-responders, integrating complementary interventions targeting neurophysiological deficits—such as neurocognitive rehabilitation (85), personalised pharmacological interventions (86), lifestyle modifications (87), and ERP-guided neurofeedback protocols (88)—may enhance treatment outcomes. Incorporating pre-treatment SPWd screening and comprehensive baseline assessments could further facilitate the early identification of individuals who may benefit from tailored or extended therapeutic approaches. Validating these strategies through controlled research is crucial to optimise their efficacy. This multimodal framework aligns with the growing emphasis on personalised mental health care, advocating for the integration of biological, psychological, cognitive, and behavioural data to maximise therapeutic impact and improve client outcomes.

### P3d as a neurophysiological index of treatment response

The findings of this study underscore the potential role of neurophysiological changes, specifically changes in the amplitude of the P3d wave, in indexing therapeutic improvements in trauma-related symptoms as measured by the Harvard Trauma Questionnaire (HTQ). The PLSC analysis demonstrated a significant multivariate association between increased P3 amplitudes and reductions in individual HTQ symptom scores, with the strongest effects observed across multiple hyperarousal symptoms, including feeling jumpy or easily startled, difficulty sleeping, and being on guard. In addition, significant associations were observed for one avoidance symptom (avoiding activities that serve as trauma reminders) and one intrusion symptom (feeling as though the traumatic event is happening again). These findings suggest that the P3d wave may serve as a promising neuromarker for understanding and monitoring treatment response in neurofeedback therapy.

The positive correlation between P3d amplitude and change in clinical symptoms (**Figure 6**) provides preliminary support for the hypothesis that changes in the P3d wave may be linked to improvements in trauma-related symptoms. Given that the P3d wave is associated with cognitive control and attentional regulation—processes frequently disrupted in individuals with PTSD—the observed increase in P3 amplitude following neurofeedback treatment could suggest potential enhancements in these capacities. However, while these findings are consistent with the idea that such changes might contribute to reductions in hyperarousal and overall symptom severity, further research is necessary to establish a definitive causal relationship and to clarify the mechanisms underlying these observations.

It is important to note that while the strongest association was observed between increased P3 amplitudes and reductions in hyperarousal symptoms, the most significant overall symptom reduction pre- to post-NFT occurred in dysphoric arousal symptoms related to functional impairment. A significant overall reduction in dysphoric arousal symptoms, including difficulties with concentration, anger, and sleep, could suggest a broader influence of neurofeedback on daily functioning. These broader effects may reflect the potential extension of improved cognitive control to integrative processes that support functional recovery in daily life, though this requires further investigation. While these interpretations offer a preliminary framework for understanding the multidimensional effects of NFT, additional research is essential to clarify the mechanisms driving these changes and their differential impact on PTSD symptom clusters. Future studies might benefit from combining connectivity analyses (e.g., coherence, phase synchrony) with graph-theoretical measures to better understand the network organisation underlying symptom clusters such as dysphoric arousal.

These results also provide valuable insights into the variability of treatment response. While the analysis included all participants, the continuum of changes observed suggests that treatment outcomes may not always align with an arbitrary binary responder/non-responder classification. Instead, the data point to the advantage of measuring change dimensionally to provide a better understanding of individual responses to neurofeedback therapy.

However, while the statistical significance of the results (p-value <0.05) strengthens confidence that these relationships are unlikely due to chance, caution is warranted in interpreting the findings. The use of difference scores highlights the dynamic nature of these changes, but it also introduces complexity in attributing causality. Additionally, the mechanisms linking neurophysiological changes to symptom reduction remain speculative, requiring further controlled and longitudinal studies to confirm the robustness and generalisability of these effects.

While these findings are promising, they should be considered within the broader context of neurofeedback research and clinical practice, emphasising the need for replication and further exploration of mediating factors that contribute to therapeutic outcomes.

### Study limitations and directions for future research

This study's use of data from routine clinical practice from a single centre, while enhancing real-world relevance, introduces limitations. The absence of a randomised control group prevents definitive attribution of observed improvements to neurofeedback therapy. Randomised controlled trials are needed to establish causal relationships and isolate NFT-specific effects. Classifying clients into responders and non-responders, reduces statistical power due to smaller subgroup sizes. Future research should prioritise larger, more balanced samples to increase statistical robustness and generalisability. This would be best achieved by a coordinated multi-centre research effort.

Additionally, the absence of clinician-administered diagnostic interviews—such as the Clinician-Administered PTSD Scale (CAPS), widely considered the gold standard for PTSD assessment—is acknowledged as a limitation of this study. However, the use of CAPS was not feasible within our clinical context, given the multicultural and multilingual nature of our participant population. Administering the CAPS with interpreters would have substantially increased assessment time, placing additional burden on participants and potentially compromising the reliability and consistency of the data. Moreover, the CAPS could not be incorporated into the computerised platform used to deliver assessments uniformly across participants. While the Harvard Trauma Questionnaire (HTQ), Version 4, offered a validated, accessible, and culturally adapted self-report alternative, the exclusive reliance on self-report measures may limit the diagnostic precision of PTSD classification in this sample.

The additional limitation of this study is the inability to systematically assess participants' ability to modulate neurofeedback training (NFT) targets over time. Conducted in a real-world clinical setting, the study employed flexible and individualised NFT protocols, often combining different approaches within a single session and adjusting reward frequencies based on participant needs. While clinically appropriate, this variability limited our ability to analyse NFT target modulation in a consistent and measurable way. As a result, we were also unable to investigate whether responders and non-responders differed in their capacity to modulate NFT targets—an analysis that could have provided a valuable insight into the ERP baseline and post-treatment differences between these groups. Future studies should consider using more standardised NFT protocols and incorporating detailed session-level data capture to allow for evaluation of target modulation and its potential role in differentiating treatment responders from non-responders. Incorporating real-time performance metrics and stratifying participants based on modulation success may also provide deeper insight into the neurophysiological mechanisms underlying treatment responsiveness.

One limitation of this study is the use of independently collected control ERP and behavioural data, which may introduce variability unrelated to the intervention. To minimise this variability, both the treatment and control groups were assessed using the same EEG hardware, software, and data acquisition procedures, thereby enhancing methodological consistency and supporting comparability of ERP measures. Nevertheless, the use of a separately collected dataset limits the strength of direct comparisons. Future studies should aim to include prospectively recruited and matched control groups assessed under identical conditions to ensure greater internal validity.

An active control group would help clarify the predictive capacity of ERPs for NFT response compared to spontaneous remission. Designing an appropriate control condition is essential to isolate the specific effects of neurofeedback therapy (NFT). Future studies should consider incorporating a sham neurofeedback control group, where participants receive feedback that is not contingent on their own brain activity. This design would help control for expectancy effects, therapist interaction, and engagement with the neurofeedback interface. Alternatively, waitlist controls or treatment-as-usual groups (e.g., trauma counselling only) could be used to evaluate the additive benefits of NFT. However, ethical considerations in withholding active treatment from severely symptomatic refugee populations must be carefully addressed. A multi-centre trial design would also be required, as recruiting for a randomised, blinded trial would be particularly challenging due to trust-related issues associated with the traumatic experiences of organised violence endured by this particular group. Nonetheless, a randomised controlled trial (RCT) combining these designs and stratified by symptom severity would strengthen causal inference and enable evaluation of both specific and non-specific treatment effects. Including ERP measures at pre-, post-, and follow-up time points would also allow for assessment of whether neurophysiological changes are sustained over time and uniquely attributable to NFT. Furthermore, as ERPs were assessed only at the post-treatment stage in the present study, future research should replicate this approach with extended follow-up assessments. Although all clients in this study received trauma counselling, direct comparison with trauma-focussed CBT would help clarify the role of NFT in the treatment of chronic PTSD.

Beyond sample and design limitations, methodological transparency represents another area for improvement. A key limitation of this study is the lack of preregistration and its retrospective design. Without preregistration of hypotheses, methods, and outcome measures, there is an increased risk of reporting and outcome bias (89). While preregistration is not yet commonly implemented in clinical neurofeedback studies, future studies should make use of templates specific to EEG/ERP research that are now available (90, 91) and the recommendations for transparent reporting of ERP studies (92), which offer structured guidance for preregistration and open science practices. Preregistration is also listed as a core recommendation in the CRED-nf checklist (37). Formats such as Registered Reports (RRs)—which involve peer review of the study protocol prior to data collection and offer publication irrespective of outcome—may also help to reduce bias and support methodological transparency (93, 94). We acknowledge that the study deviates from several CRED-nf checklist recommendations, including the absence of preregistration, a control condition, and blinding. These limitations are partly due to the retrospective and naturalistic nature of the study design but should be addressed in future trials.

While the results demonstrate a significant association between increased P3 amplitudes and reductions in HTQ scores, this does not establish causation. Other factors, such as therapeutic alliance, concurrent treatments, or participants' inherent neuroplasticity, could contribute to these changes.

Neurofeedback therapy may have broader systemic effects that are not related to P3 amplitude changes, requiring additional controls or cross-validation.

A further limitation of the current study is the absence of source localisation analyses. Due to the use of a low-density EEG system and the lack of individual anatomical data, we were unable to perform beamforming or other source reconstruction methods to directly infer the cortical origins of the observed ERP changes. While our scalp-level findings provide important information, they do not allow for precise mapping of the underlying brain regions involved. Future studies employing high-density EEG and/or multimodal imaging could more accurately localise neural activity and enhance the interpretability of ERP results. Incorporating additional neuroimaging techniques, such as fMRI, alongside ERPs could offer a more comprehensive understanding of treatment effects by clarifying the underlying neural mechanisms.

While ERP markers such as P3d and SPW amplitudes were used to assess neurocognitive changes, the functional role of the SPW in PTSD, remains unclear and merits further investigation. These gaps highlight the need for studies exploring the specific cognitive and affective processes linked to SPW alterations in PTSD.

The observation of a continuum in treatment response, where individuals with greater improvements in P3 amplitudes had more significant reduction in HTQ scores, highlights the variability in NFT outcomes. While the results suggest that increased P3 amplitudes are associated with better outcomes, it remains unclear whether these changes are a direct result of NFT or reflect broader therapeutic processes.

Several clinically relevant factors, such as comorbidities, chronic physical illnesses, confirmed traumatic brain injury (including post-concussion syndrome), and variations in counselling practices, were not accounted for due to the nature of data collection and the heterogeneity of the sample. While statistically controlled, differences in the amount of trauma counselling received could also have influenced outcomes. Although responders were more likely to be medicated, our sensitivity analysis indicated that medication status did not significantly predict symptom change, and the observed treatment effects remained robust after accounting for this factor. Clients were prescribed a wide variety of medications by external providers, limiting our ability to draw conclusions about the effects of specific pharmacological agents. Controlled studies are needed to explore potential synergistic or augmenting effects of neurofeedback when combined with psychotropic medication. Another limitation is the absence of direct assessment of dissociative symptoms, despite their known prevalence in trauma-exposed refugee populations and potential influence on neurofeedback engagement and neural outcomes. The dissociative subtype of PTSD, often characterised by emotional numbing and altered brain connectivity, may partially explain the heterogeneity in treatment response and ERP profiles observed in this study. Future research should incorporate validated dissociation measures (e.g., DES, MID) and examine whether dissociative symptomatology moderates neurofeedback outcomes or ERP changes, particularly in samples with significant early life trauma.

Future studies should address these limitations through more rigorous methodological designs to strengthen and expand upon the current findings.

Future research should also try to test standardised NFT protocols for specific behavioural deficits such as inhibitory control. Investigating correlations between improvements in these areas and changes in ERP markers (e.g., P3d, SPW amplitudes) could validate their utility as indicators of neuroplasticity and treatment progress. Longitudinal studies assessing the stability of these improvements and their relationship to functional recovery in PTSD populations are crucial.

Incorporating concurrent EEG and neuroimaging (e.g., fMRI) may uncover additional predictors of treatment efficacy. Examining long-term effects and impact across diverse PTSD populations (varying trauma backgrounds, symptom severity) will provide crucial insights into generalisability and applicability.

Finally, to address the challenges of cost-effectiveness and scalability in neurofeedback interventions, home-based and mobile EEG neurofeedback approaches merit further exploration. Cooke et al. (95) recently demonstrated the feasibility of remotely delivering EEG neurofeedback in clinical populations, offering a robust model for scalable and rigorously designed interventions. Their recommendations—including preregistration, adherence to the CRED-nf checklist, and the incorporation of wearable EEG neurofeedback technologies—provide a useful framework for expanding access while preserving methodological integrity.

Growing evidence supports the clinical utility of mobile EEG neurofeedback across diverse and underserved populations. For instance, du Bois et al. (96) reported significant reductions in PTSD symptoms following the use of low-cost, wearable EEG neurofeedback in community-based settings. Similarly, Elbogen et al. (97) found that mobile neurofeedback effectively reduced chronic pain in veterans with TBI and PTSD, reinforcing the potential for remote, cost-effective treatment delivery.

Nevertheless, trauma-focussed neurofeedback requires careful adaptation. The involvement of a trained clinician remains essential—not only to ensure safe application but also to facilitate therapeutic integration with trauma counselling. As Tsuji-Lyons and White (98) emphasised, clinician-guided neurofeedback fosters emotional safety and supports the processing of complex trauma. Accordingly, while mobile neurofeedback offers an important avenue for broadening access—especially in remote or low-resource contexts—hybrid delivery models that combine clinical oversight with technological flexibility may offer the most ethically sound and effective approach for trauma-affected populations.

## Conclusion

NFT shows promise as a targeted intervention for addressing cognitive control deficits and alleviating PTSD symptoms in refugees with chronic treatment-resistant PTSD. Responders to NFT showed significant post-treatment improvements in P3d amplitudes, a marker of enhanced inhibition control and attentional resource allocation, while the SPWd amplitude, which differentiated responders from non-responders at baseline, did not change with treatment. The normalisation of P3d amplitudes in responders highlights the potential of ERP markers as indicators of treatment progress, providing a valuable framework for monitoring and refining therapeutic interventions. These insights pave the way for future research to refine NFT protocols, optimise treatment outcomes, and enhance recovery for individuals living with chronic treatment-resistant PTSD, particularly those from vulnerable and complex trauma populations.

Despite its promise, the study is limited by the lack of a control group, and that it was conducted at a single centre. Future research should adopt larger, controlled studies and explore the functional role of P3d and SPWd in PTSD.

## Data Availability

The raw data supporting the conclusions of this article will be made available by the authors, without undue reservation.
