# Supplementary figures and images for: Enhanced cognitive control following neurofeedback therapy in chronic treatment-resistant PTSD among refugees: a feasibility study
Source: Front Psychiatry. 2025 Aug 15;16:1567809. doi: 10.3389/fpsyt.2025.1567809 (PMC12394473; doi:10.3389/fpsyt.2025.1567809)

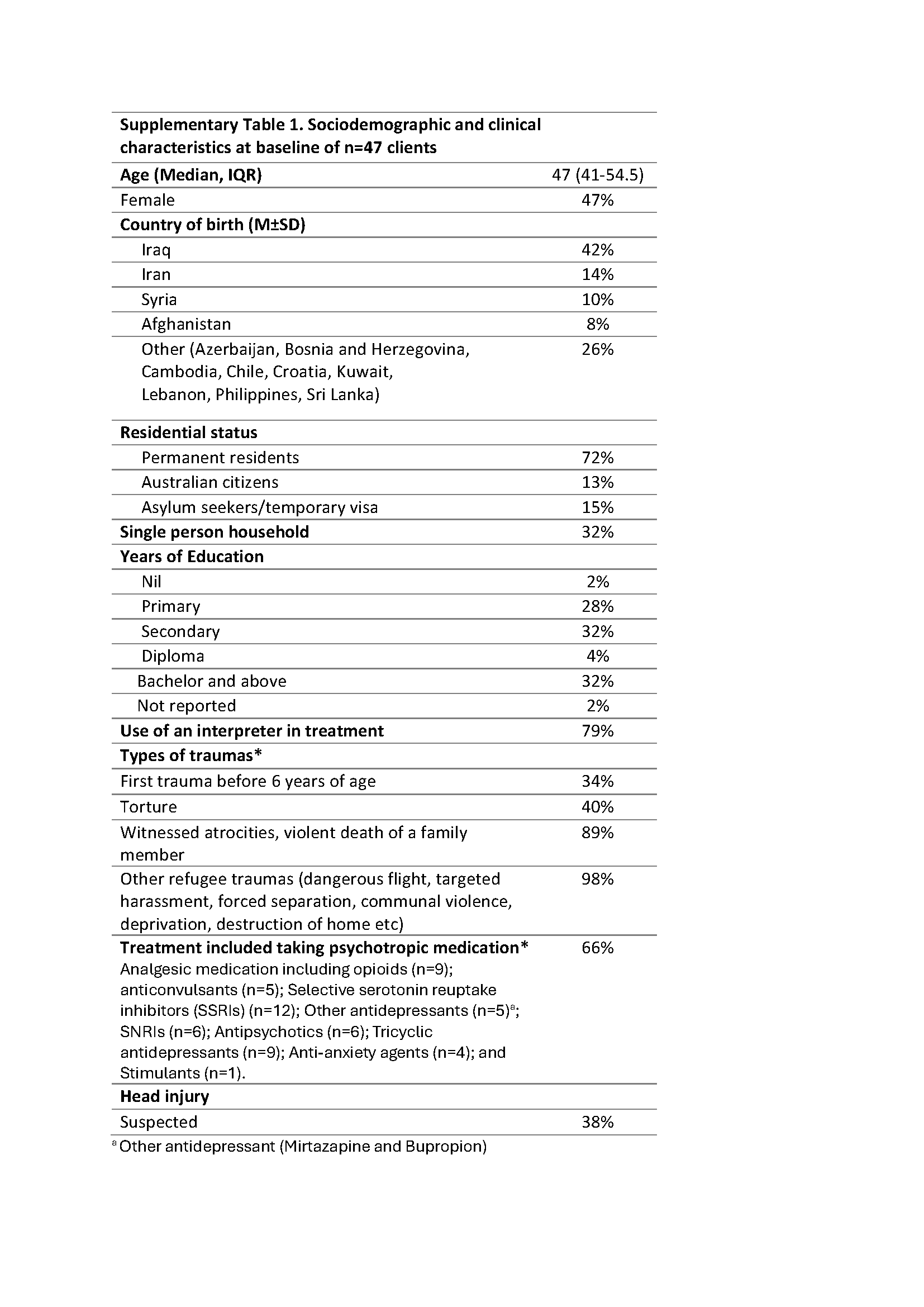

Supplement: Supplementary file 1 [file Image1.tiff]

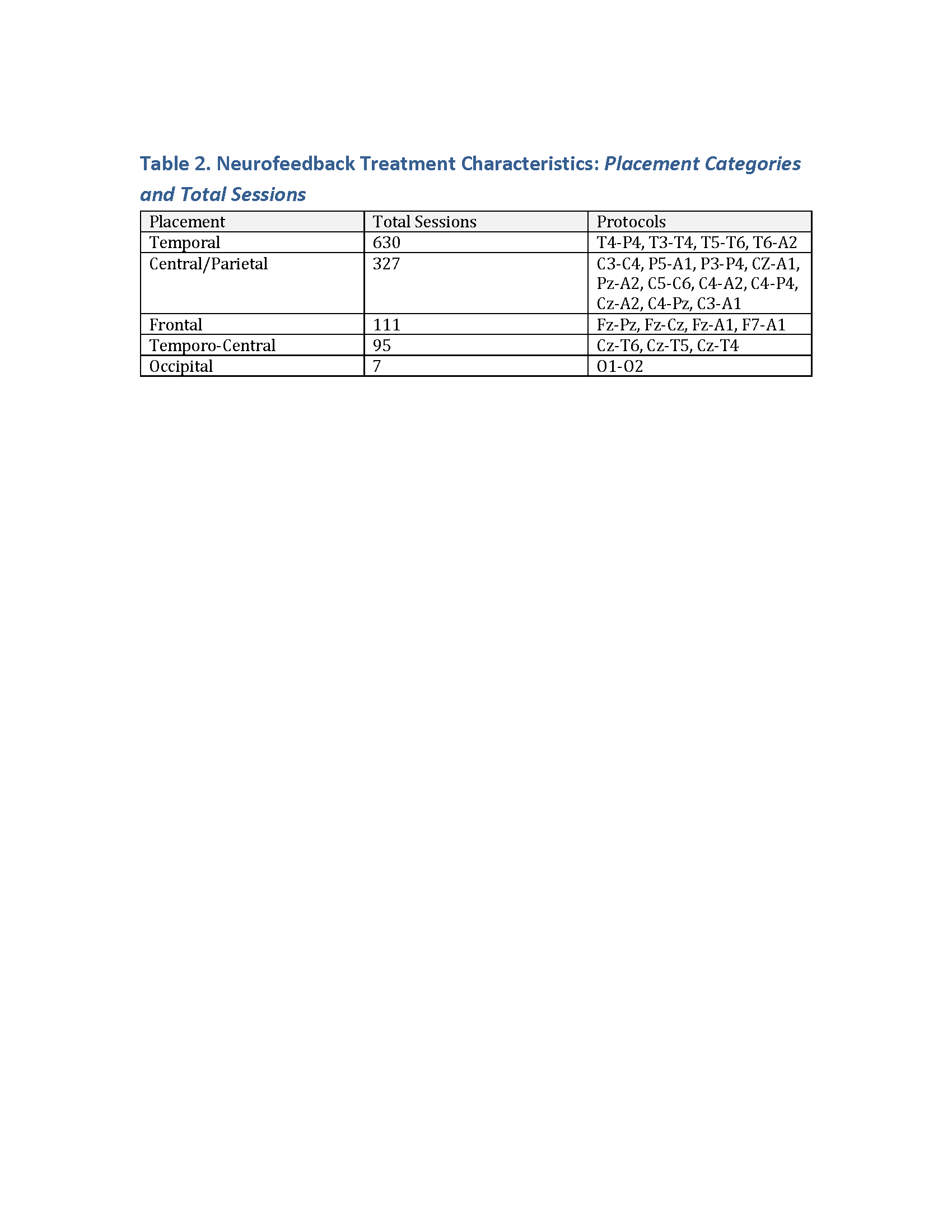

Supplement: Supplementary file 2 [file Image2.tiff]
