# Supplementary material for: Enhanced cognitive control following neurofeedback therapy in chronic treatment-resistant PTSD among refugees: a feasibility study
Source: Front Psychiatry. 2025 Aug 15;16:1567809. doi: 10.3389/fpsyt.2025.1567809 (PMC12394473; doi:10.3389/fpsyt.2025.1567809)
Supplement: Supplementary file 3 [file Table3.pdf]

CRED-nf Checklist (Post Hoc Completion)

Manuscript title: Enhanced Cognitive Control Following Neurofeedback Therapy in Chronic Treatment-Resistant PTSD Among Refugees: A Feasibility Study

Corresponding author: Mirjana Askovic

Email: mirjana.askovic@health.nsw.gov.au

Section 1: Pre-experiment

Item No.	Checklist Item	Manuscript Details
1a	Pre-register experimental protocol and planned analyses	Not preregistered; retrospective design. Addressed as a limitation.
1b	Justify sample size	Feasibility study; sample size determined by available clinical cases. Now discussed with reference to Lakens (2022).

Section 2: Control Groups

Item No.	Checklist Item	Manuscript Details
2a	Employ control group(s) or control condition(s)	No control group; naturalistic, one-arm feasibility study. Comparison made with normative ERP dataset.
2b	Double-blind design (when appropriate)	Not applicable.
2c	Blind outcome raters and data analysts	Not blinded; acknowledged as a limitation.
2d	Examine maintenance of blinding	Not applicable.
2e	Use standard-of-care control in clinical efficacy trials	Not a clinical efficacy trial.

Section 3: Control Measures

Item No.	Checklist Item	Manuscript Details
3a	Collect psychosocial factors	Limited to demographic and trauma history variables.
3b	Report whether participants were provided with strategies	Strategies were not standardised; neurofeedback protocols adjusted based on clinician assessment.
3c	Report participant-reported strategies	Not recorded.
3d	Report online data processing and artefact correction	Artefact rejection performed; not detailed online processing.
3e	Report condition/group effects for artefacts	Not assessed separately; described generally.

Section 4: Feedback Specifications

Item No.	Checklist Item	Manuscript Details
4a	Define online feature extraction	Delta/Theta, Alpha and beta bands used; visual and auditory feedback provided via EEGer software.
4b	Justify reinforcement schedule	Reinforcement threshold adjusted manually (target ~60–70% success).
4c	Report feedback modality and content	Visual and auditory; e.g., "Formation" game used as feedback.
4d	Report feedback variables or contrasts shown to participants	Inhibit/reward band amplitudes.
4e	Report hardware/software	EEGer-4 software, J&J Spectrum 4-channel

		amplifier. Reported in Methods.

Section 5: Outcome Measures – Brain

Item No.	Checklist Item	Manuscript Details
5a	Report neurofeedback regulation success	Not quantified in-session regulation success.
5b	Plot within-/between-session feedback changes	Not included; acknowledged as a limitation.
5c	Compare experimental group to control group	No control group; comparison to healthy ERP dataset (n=107).

Section 6: Outcome Measures – Behaviour

Item No.	Checklist Item	Manuscript Details
6a	Include a priori definitions of clinical/behavioural significance	Responders defined as ≥0.5 point HTQ improvement.
6b	Correlate regulation success and behavioural outcomes	Not applicable; regulation success not quantified.

Section 7: Data Transparency

Item No.	Checklist Item	Manuscript Details
7a	Upload materials, analysis code, and data to open repository	Not uploaded due to retrospective design and ethical/privacy considerations. Available upon reasonable request.

Note: This CRED-nf checklist (Ros et al., 2020) has been completed post hoc to enhance transparency in reporting the neurofeedback components of the study. Deviations from checklist recommendations are acknowledged in the Limitations section.
